# Basin-scale study of CO_2_ storage in stacked sequence of geological formations

**DOI:** 10.1038/s41598-024-66272-x

**Published:** 2024-08-12

**Authors:** Nur Wijaya, David Morgan, Derek Vikara, Timothy Grant, Luciane Cunha, Guoxiang Liu

**Affiliations:** 1 National Energy Technology Laboratory (NETL) Support Contractor, 626 Cochrans Mill Road, P.O. Box 10940, Pittsburgh, PA 15236 USA; 2 NETL, 626 Cochrans Mill Road, P.O. Box 10940, Pittsburgh, PA 15236 USA; 3 Repsol USA, 2455 Technology Forest Blvd, The Woodlands, TX 77381 USA

**Keywords:** Carbon capture and storage, Basin-scale modeling, Saline formations, Pressure buildup, CO_2_ plume commingling, Energy science and technology, Carbon capture and storage

## Abstract

Commercial scale decarbonization through carbon capture and storage may likely involve many CO_2_ storage projects located in close proximity. The close proximity could raise concerns over caprock integrity associated with reservoir pressure buildup and interference among adjacent projects. Commercial-scale injection will also require large prospective CO_2_ storage resource and high injectivity in the targeted storage formations. To accommodate the need for both large resource and high injectivity, project operators could consider injecting CO_2_ into a stacked sequence of formations. This analysis investigates the benefits of injecting CO_2_ into a vertically stacked sequence of saline formations, over injecting the same amount of CO_2_ into a single saline formation, in addressing these challenges. Our analysis shows that injecting into the stacked sequence mitigates the extent of pressure buildup among the stacked formations, while still achieving the same or greater target CO_2_ storage volumes. Among cases modeled, the resulting pressure buildup front is most reduced when each storage site distributes injection volumes over several wells, each of which injects a portion of the total CO_2_ mass across the stacked sequence. This favorable case not only results in the smallest CO_2_ aerial footprint, but also shows the largest reduction in the pressure buildup at the top of perforation at the injection wells (upwards of approximately 46% compared to the single-formation storage), the result of which is crucial to maintain caprock integrity. This analysis provides insights into required decision-making when considering multi-project deployment in a shared basin.

## Introduction

Carbon capture and storage (CCS) has been proposed as a key decarbonation strategy to control the climate change by capturing anthropogenic carbon dioxide (CO_2_) from stationary CO_2_ emitters and storing it permanently in underground geologic formations^1–6^. The target injection zones mainly consist of three types: saline formations^7^, depleted oil and gas reservoirs^8,9^, and unmineable coal beds^10,11^. Among these, saline formations are identified as promising CO_2_ injection option^12,13^ because they offer the greatest prospective CO_2_ storage resource, which is defined as the mass of CO_2_ that can theoretically be stored in available pore space at the primary stage of a CO_2_ storage project^14–17^. Saline formations are brine-saturated porous and permeable rocks with total dissolved solids (TDS) higher than the cutoff for protected underground sources of drinking water (USDW), which is generally 10,000 parts per million (ppm) in the United States (U.S.)^18–20^.

The U.S. Department of Energy (DOE)’s National Energy Technology Laboratory (NETL) estimated between 2379 and 21,633 billion metric tons of prospective CO_2_ storage resource in saline formations in onshore North America based on the onshore CO_2_ volumetric assessment effort under the Regional Carbon Storage Partnerships (RCSPs)^21–23^. Through this effort, it was demonstrated that saline formations are widely distributed across onshore North America and co-located with major CO_2_ stationary emitters^24^, the setting of which offers a unique opportunity for the decarbonization goals. To foster the deployment of large-scale CO_2_ storage projects, the U.S. DOE NETL launched the Carbon Storage Assurance Facility Enterprise (CarbonSAFE) Initiatives, which aim to build off the work done by the RCSPs to fund and develop more projects. More importantly, these projects provide lessons learned for commercial-scale CCS projects, which are defined as those in which at least 50 million metric tons (Mt) of CO_2_ are injected underground over the course of next 20 to 30 years^25^.

### Close proximity among injection wells

To accommodate the extensive target injection rates, the commercial-scale CCS projects may likely involve many CO_2_ storage projects located in close proximity. The commercial-scale injection may create pressure buildup in the storage formations, the magnitude of which depends on the volumetric size and hydraulic conductivity of storage formations. In this analysis, the pressure buildup is defined as

1 $$\Delta P\left(x,y,z,t\right)=P\left(x,y,z,t\right)-{P}_{i}(x,y,z)$$

where $\Delta P\left(x,y,z,t\right)$ is the pressure buildup at a given point ($x,y,z$) in the formation at time $t$, $P\left(x,y,z,t\right)$ is the instantaneous pressure existing at the corresponding point ($x,y,z$) at the instant $t$ considered, and ${P}_{i}(x,y,z)$ is the initial ambient reservoir pressure at the corresponding point ($x,y,z$). In the absence of basin-wide pressure management operations (e.g., brine production from the storage formations), the pressure buildup can be extensive, which would limit the amount of CO_2_ that can be safely stored in the storage formations. The U.S. Environmental Protection Agency (EPA) regulates underground injection activities in order to prevent contamination of current and future USDW through its Underground Injection Control (UIC) program^26^. CO_2_ injection for long-term storage is a practice subject to UIC rules under Class VI, which are noted in the Code of Federal Regulations (CFR) parts 40 CFR 146.81–40 CFR 146.95. The U.S. EPA Class VI rules stipulate that the maximum reservoir pressure in the injection zone cannot exceed 90% of fracture pressure^26^, which this analysis defines as “fracture pressure threshold” given by

2 $$\gamma \left(x,y,z\right)={0.9\times P}_{f}\left(x,y,z\right)-{P}_{i}\left(x,y,z\right)$$

where $\gamma \left(x,y,z\right)$ is the fracture pressure threshold at a given point ($x,y,z$) in the formation and ${P}_{f}\left(x,y,z\right)$ is the fracture pressure at the corresponding point ($x,y,z$). Assuming a homogeneous fracture pressure gradient ($\nabla {P}_{f}$ presented in psi/ft in this analysis) across a particular area, as assumed in this analysis, $\gamma \left(x,y,z\right)$ is simplified to $\gamma \left(z\right)$. This fracture pressure threshold ($\gamma$) serves as a critical constraint to avoid large-scale pressure buildup to maintain caprock integrity, avoid any potential existing fault impact, significant stress perturbations, and prevent brine and/or CO_2_ from leaking into the overlying USDW^27–29^. Excessive pressure buildup can cause the water table to rise, which could increase the rates of water discharge into surface lakes or streams^30,31^ and change surface and subsurface flow patterns from land-surface deformation or uplift^31^.

The close proximity among storage projects may create large pressure interference early in the injection period; this can amplify the magnitude of pressure buildup, especially near the injection wells. Pressure interference is defined in this analysis by:

3 $${\epsilon }_{i-j}\left(x,y,z,t\right)={\Delta P}_{i}\left(x,y,z,t\right)-{\Delta P}_{j}\left(x,y,z,t\right)$$

where ${\epsilon }_{ij}\left(x,y,z,t\right)$ is the pressure interference at a given point ($x,y,z$) in the formation at time $t$, ${\Delta P}_{i}\left(x,y,z,t\right)$ is the pressure buildup of a given case $i$ at the corresponding point ($x,y,z$) at the instant $t$ considered, and ${\Delta P}_{j}\left(x,y,z,t\right)$ is the pressure buildup of the baseline case $j$ at the corresponding point ($x,y,z$) at the instant $t$ considered. Pressure interference could result in more rapid pressure buildup and limit the injection rate. Consequently, the anticipated volume of captured CO_2_ could require other operational strategies (e.g., additional sink options) to store.

Additionally, the close proximity would require another consideration associated with the near-site extent of CO_2_ plume migration. If the sites are managed by the same operator, CO_2_ plume commingling among the sites is desirable because it promotes efficient use of the subsurface pore space. Otherwise, CO_2_ plume commingling could raise concerns regarding long-term legal liability among different storage operators in the event of potential leakage of commingling CO_2_ plumes.

### Large prospective CO_2_ storage resource and high injectivity

The basin-wide target injection rates of commercial-scale CCS projects are large, sometimes in the range of hundreds of millions of metric tons of CO_2_ each year (i.e., hundreds of Mt/year)^32–35^. Therefore, the commercial-scale CCS projects require large prospective CO_2_ storage resource and high formation injectivity^36,37^. To accommodate the need for both, project operators could consider injecting CO_2_ into a stacked sequence of formations. Injection approaches distributed across stacked sequences are perceived to reduce the pressure buildup and interference among injection wells that may otherwise occur with injection into a single formation.

**Figure 1:**
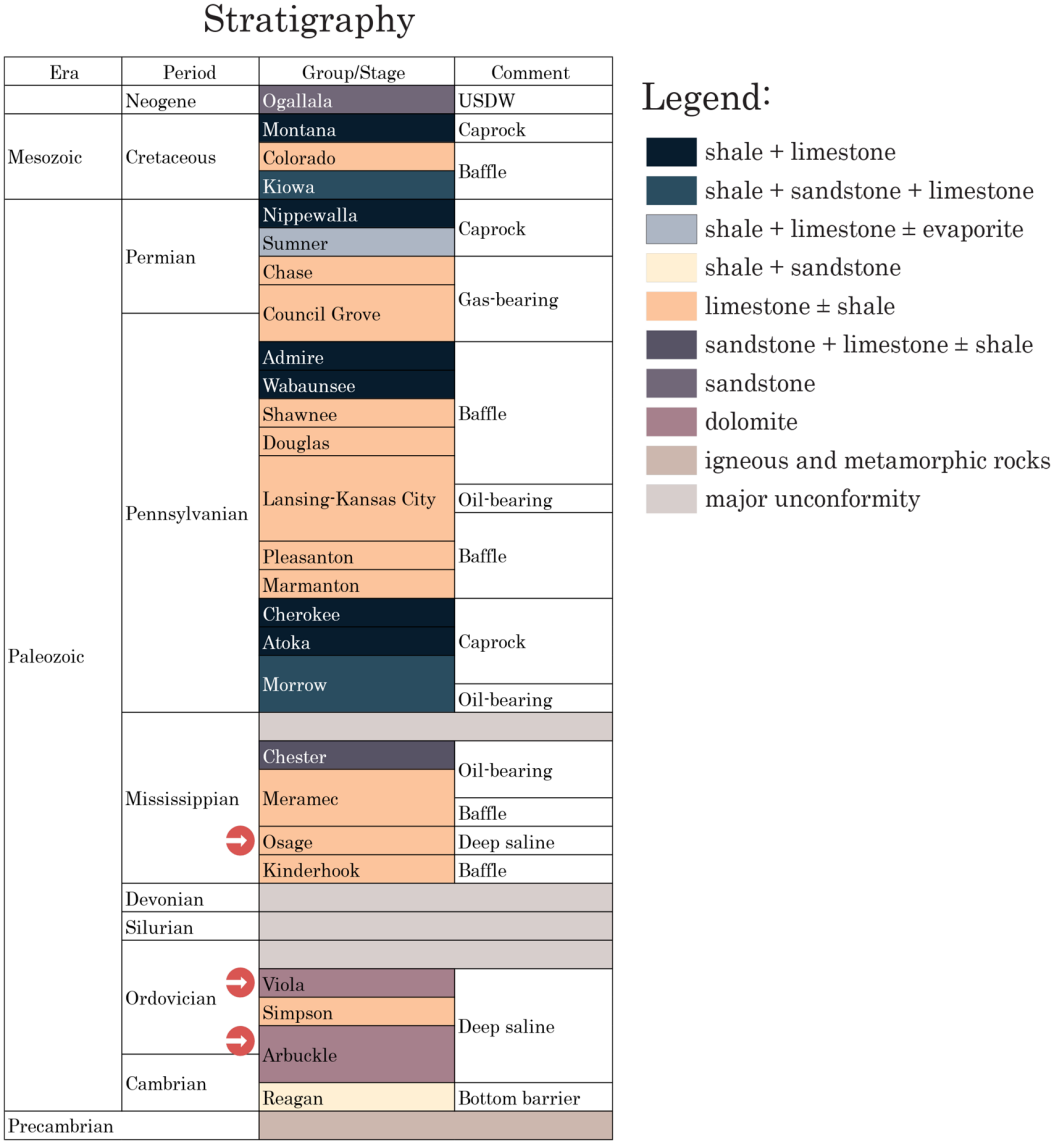
Generalized stratigraphic chart for Patterson site in southwest Kansas considered in IMSCS-HUB CarbonSAFE project modified from Holubnyak et al.^39^.

For instance, as part of the CarbonSAFE Initiatives, the Phase II of Integrated Midcontinent Stacked Carbon Storage Hub (IMSCS-HUB) project evaluates the potential of CO_2_ storage into a stacked sequence in the states of Kansas and Nebraska in the U.S^38^. One of its target storage hubs is the Patterson site in Kansas, U.S.^39^. This site is planned to target a vertically stacked sequence of three saline formations (in decreasing depth): Arbuckle (Cambrian-Ordovician), Viola (Ordovician), and Osage zones (Mississippian) that are vertically isolated by thick and tight carbonates and thin shale intervals serving as aquitards (Fig. 1).

To evaluate the prospective CO_2_ storage resource in the Patterson site, the U.S. DOE methodology known as NETL CO_2_-SCREEN was used^39,40^. To consider injectivity and pressure limitation for storage, Leng et al. reported some valuable efforts based on analytical simulation tools^41–43^. Tables 1 and 2 summarize their estimates on the geologic properties and probabilistic prospective CO_2_ storage resource of the Patterson site, respectively. Table 2 shows that if the commercial-scale storage project only targets a single formation, only the Arbuckle formation seems to demonstrate sufficient prospective CO_2_ storage resource, regardless of the geological realization of variations in the CO_2_ storage efficiency^44^. In other words, CO_2_ injection into the stacked sequence of the three formations is necessary to maximize the potential use of pore spaces present in the Viola and Osage formation.

**Table 1:** Estimated geologic properties in the Patterson site^39^.

Parameter	Unit	Arbuckle	Viola	Osage
Area (mean)	km^2^	130	130	130
Gross thickness (mean)	m	174	55	46
Total porosity (mean)	%	7	7	12
Pressure (mean)	MPa	11.7	11.5	11.4
Temperature (mean)	°C	58	56	54
Lithology	–	Dolomite	Dolomite	Limestone
Storage efficiency—P10	%	8.5	9.5	11
Storage efficiency—P50	%	14	16	19.5
Storage efficiency—P90	%	21	23	29

**Table 2:** Estimated prospective CO_2_ storage resource in the Patterson site^39^.

Prospective CO_2_ Storage Resource	Unit	Arbuckle	Viola	Osage	Sum
P10	Mt	56	18	22	96
P50	Mt	105	28	34	167
P90	Mt	142	38	46	226

Another potential of CO_2_ storage in a stacked sequence is demonstrated in another CarbonSAFE project called Nebraska Integrated Carbon Capture and Storage Pre-Feasibility Study in western Nebraska in the U.S.^45^. The project identified a vertically stacked sequence of three potential CO_2_ storage formations (in decreasing depth): Cherokee Group (Middle Pennsylvanian), Cedar Hills (Lower Permian), and Cloverly (Lower Cretaceous). However, this study does not report the estimated prospective CO_2_ storage resource confidently because of low data availability and absence of characterization wells. Nonetheless, both CarbonSAFE projects demonstrate that the presence of stacked sequence of saline formations provides an opportunity for project operators to accommodate the need for extensive prospective CO_2_ storage resource and high injectivity.

### Comparison between CO_2_ storage in a stacked sequence and a single formation

While the IMSCS-HUB CarbonSAFE project performed an initial dynamic simulation of CO_2_ injection into a stacked sequence (i.e., Arbuckle, Viola, and Osage formation), the Nebraska Integrated Carbon Capture and Storage Pre-Feasibility Study CarbonSAFE project performed their injection simulation into a single formation (Cloverly formation) only, despite the identified availability of the stacked sequence (with Cedar Hills formation) in the project. Similarly, a previous study by Birkholzer et al.^46^ simulated a CO_2_ injection into a single formation only, although their model domain included a stacked sequence of saline formations with an assumption of the large model domain size to avoid boundary impact in the studies. Furthermore, Birkholzer et al.^46^ included one injection well only in the model,therefore, although they examined the extent of pressure buildup, the presence of multi-well pressure interference and the likelihood of CO_2_ plume commingling were not evaluated^46^. More importantly, while these previous studies included a stacked sequence of saline formations in their simulation model domain, a larger number of previous studies included a single target injection formation only in their simulation model domain^16,47–49^.

Consequently, there is currently a lack of systematic one-to-one comparative conceptual study between stacked-sequence and single-formation CO_2_ storage. This current state creates challenges in drawing instructive yet quantitative observations about the potential benefits of injecting into the stacked sequence over injecting into the single formation in accommodating the need for close proximity among storage sites, extensive subsurface prospective CO_2_ storage resource, and high formation injectivity.

This analysis systematically evaluates how the extents of pressure buildup and CO_2_ plumes evolve during injection and post-injection stages of a storage project in which commercial-scale multi-well CO_2_ injection operations located in close proximity occur simultaneously into a stacked sequence of saline formations. This analysis complements these previous studies by quantitatively investigating the benefits of injecting CO_2_ into a vertically stacked sequence of saline formations, over injecting the same amount of CO_2_ into a single saline formation. A one-to-one comparison between the stacked-sequence and single-formation storage is carried out to quantify these potential benefits in terms of management of pressure buildup and interference. Overall, this analysis also provides insights into required decision-making when considering multi-project deployment in a shared basin.

## Methodology

Reservoir numerical modeling was carried out using the TOUGH3 software^50^, with the ECO2M equation-of-state module^51^. TOUGH3 can solve fluid and heat flows of multiphase, multicomponent fluid mixtures in porous and fractured media^50^. TOUGH3 solves coupled nonlinear mass and energy conservation equations that are closed via the equation of state. The ECO2M module is developed to simulate the injection of CO_2_ into saline aquifers and the resulting coupled processing of multiphase fluid flow, heat transfer, and chemical reactions (which include partitioning of water and CO_2_ between the phases and precipitation/dissolution of solid salt). Unlike its predecessor, the ECO2N module, the ECO2M module can describe conditions in which both liquid and gaseous CO_2_-rich phases are present so that it can describe all possible phase conditions for brine-CO_2_ mixtures, including the transition between super- and sub-critical conditions and the phase change between liquid and gaseous CO_2_^50^.

### Reservoir model description

3D rectangular-block generic reservoir models were constructed with a set of assumed reservoir properties shown in Table 3. The reservoir models include a stacked sequence of two saline formations as the injection targets and consist of the following individual hydraulic unit layers (in increasing depth): upper seal, upper injection formation, middle seal, lower injection formation, and lower seal, all of which are assumed to be homogeneous. The top depth of upper seal is 940 m (below the surface); therefore, the top depth of upper injection zone is 1000 m, which is a sufficient depth for CO_2_ to be stored in a supercritical state, based on the in-situ pressure and temperature assumed in this analysis. These reservoir properties are not based on data from specific basins; however, they lie within the range observed in the literature for appraised saline storage reservoirs^52,53^. All model boundaries are closed (no-flow). The pressure propagation above the upper seal and below the lower seal is not analyzed, and the models in this analysis are run in an isothermal mode after the vertical distribution of thermophysical properties is generated from preceding steady-state initialization runs.

**Table 3:** Reservoir properties in models.

Parameter	Unit	Upper seal	Upper injection formation	Middle seal	Lower injection formation	Lower seal
Porosity	%	5	10	5	10	5
Horizontal permeability	mD	0.001	50	0.001	50	0.0001
Permeability anisotropy (k_h_/k_v_)	–	3.3	3.3	3.3	3.3	3.3
Thickness	m	60	200	60	200	20
Average initial reservoir pressure	MPa	9.91	11.2	12.6	13.9	15.0
Average initial temperature	°C	42.4	45.0	47.6	50.2	52.4
Initial brine concentration	ppm	30,000	30,000	30,000	30,000	30,000

Reservoir pressure and temperature conditions are selected to ensure that CO_2_ remains in the supercritical state in the injection zones^54,55^ because this state allows CO_2_ storage within a relatively small pore volume^56^. For illustration, at 0 °C and 1 atmospheric pressure (i.e., sub-critical gaseous state), one metric ton of CO_2_ occupies 509 m^3^,meanwhile, at supercritical conditions corresponding to a CO_2_ density of 700 kg/m^3^, the same mass of CO_2_ occupies 1.43 m^3^ or less than 6 m^3^ of rock with 30% porosity with 20% irreducible water saturation^5^. Therefore, in terms of formation depth, the injection formation typically exists at depths greater than 800 m^18,57^, depending on the regional in-situ fluid pressure and geothermal gradients proximal to candidate storage sites. While there is a great variation in the local geothermal gradients, the average temperature gradients in many sedimentary basins are approximately 25–30 °C/km. Meanwhile, the average pore pressure gradients of sedimentary rocks are generally close to the hydrostatic pressure gradient unless it is under- or over-pressure conditions, that is the pressure generated by a column of water of equal height to the depth of the pore space^5^.

### Modeling cases

**Figure 2:**
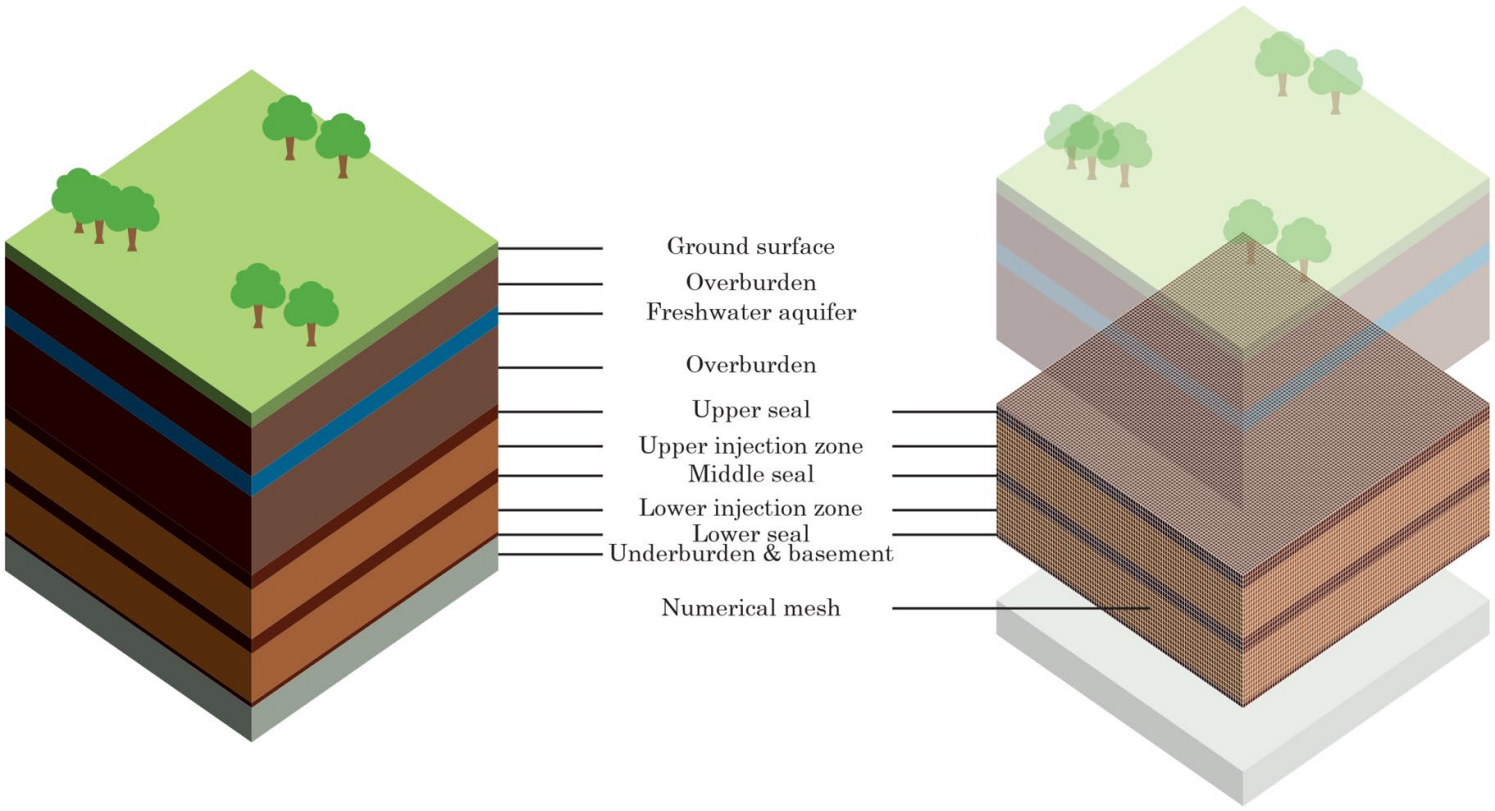
Modeling domain selected in this analysis (right) extracted from the multi-layered subsurface system (left).

**Figure 3:**
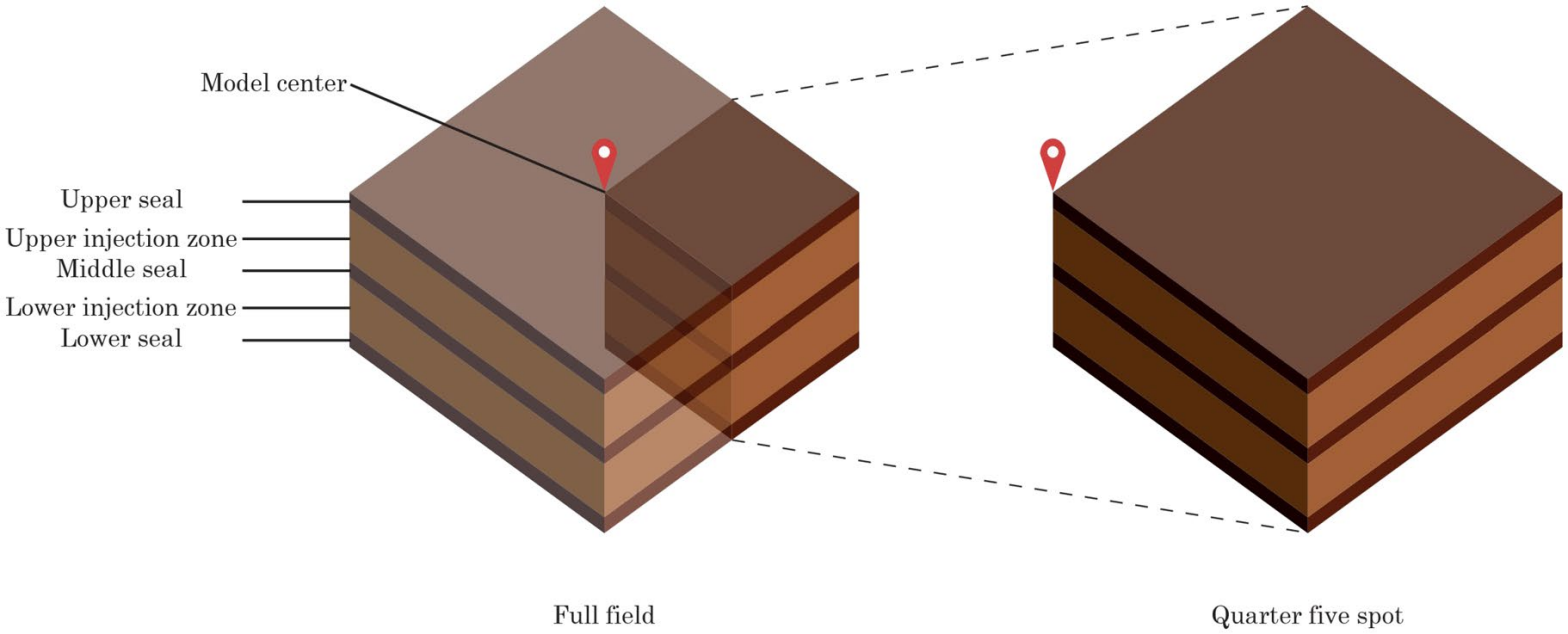
3-D schematics of quarter five spot model segment (right) extracted from the full field model (left). Figure is not drawn based on any specific scale.

Table 4 lists the modeling cases in this analysis. We begin our analysis by modeling single-site cases (Cases 1 and 2) followed by multi-site cases (Cases 3 through 5), as illustrated by the model segmentation schematics in Fig. 2. Case 1 represents the baseline case, which injects CO_2_ from a single well into a single formation. Although there are two injection wells in Case 2, both wells are located in the same storage site; therefore, Case 2 is categorized as “single-site” but in stacked upper and lower formations. The analysis on Case 1 establishes the baseline for the extent of pressure buildup and CO_2_ plume migration when there is no pressure interference among different storage sites or injection formations. Because both cases only model a single storage site, the model extent for both cases only include a quarter of a five-spot pattern in this homogenous model (Fig. 3).

**Table 4:** Modeling cases.

Case	Case group	Model segmentation	Number of injection wells in model domain	Number of target injection formations	Target injection formation	Multi-site pressure interference	Model aerial extent (km × km)
1	Single-site	Quarter five-spot	1	1 (Lower)	Single	No	500 × 500
2	Single-site	Quarter five-spot	2	2 (Lower and Upper)	Stacked	No	500 × 500
3	Multi-site	None (full field)	4	1 (Lower)	Single	Yes	1000 × 1000
4	Multi-site	None (full field)	4	2 (Lower & Upper)	Stacked	Yes	1000 × 1000
5	Multi-site	None (full field)	8	2 (Lower & Upper)	Stacked	Yes	1000 × 1000

**Figure 4:**
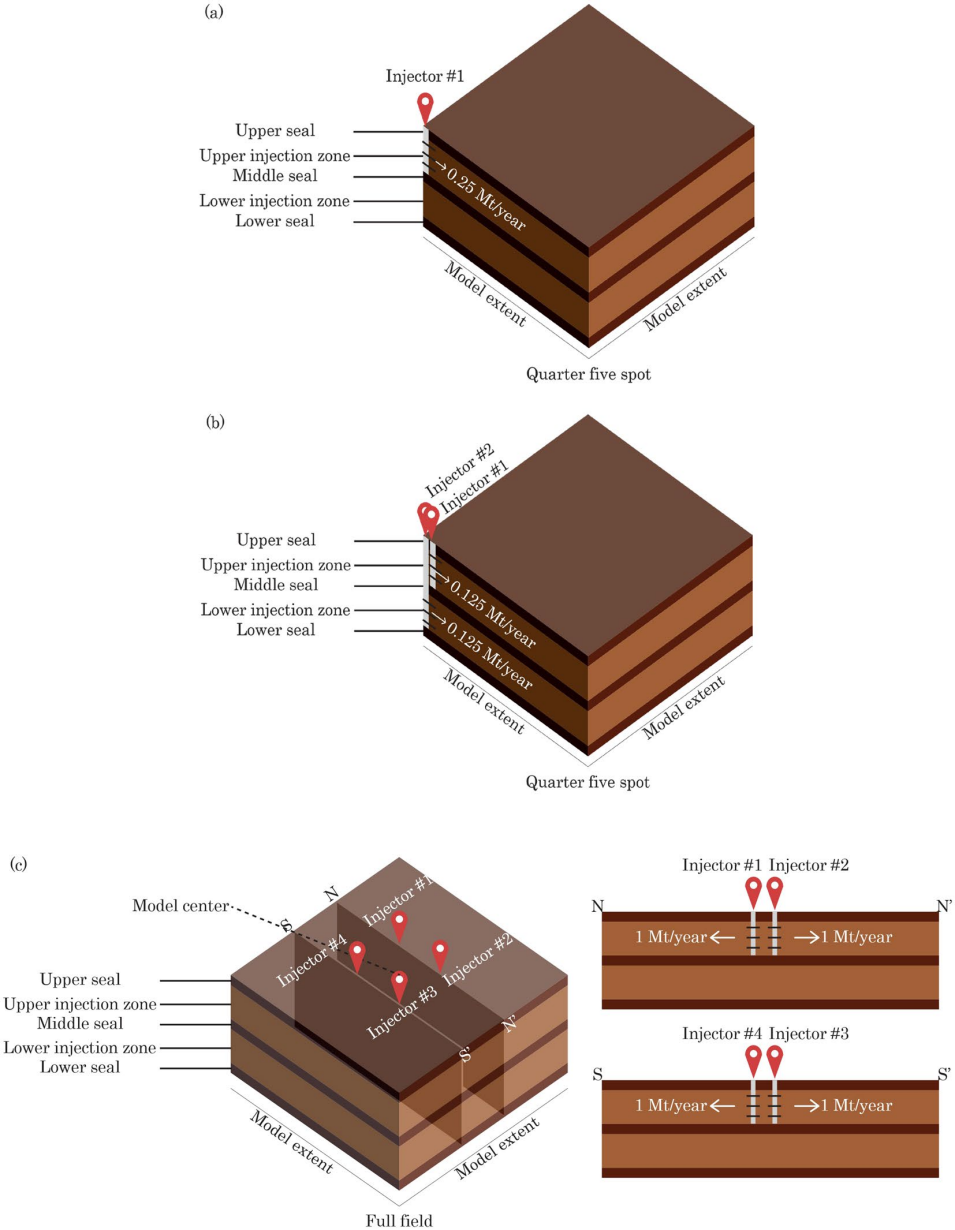

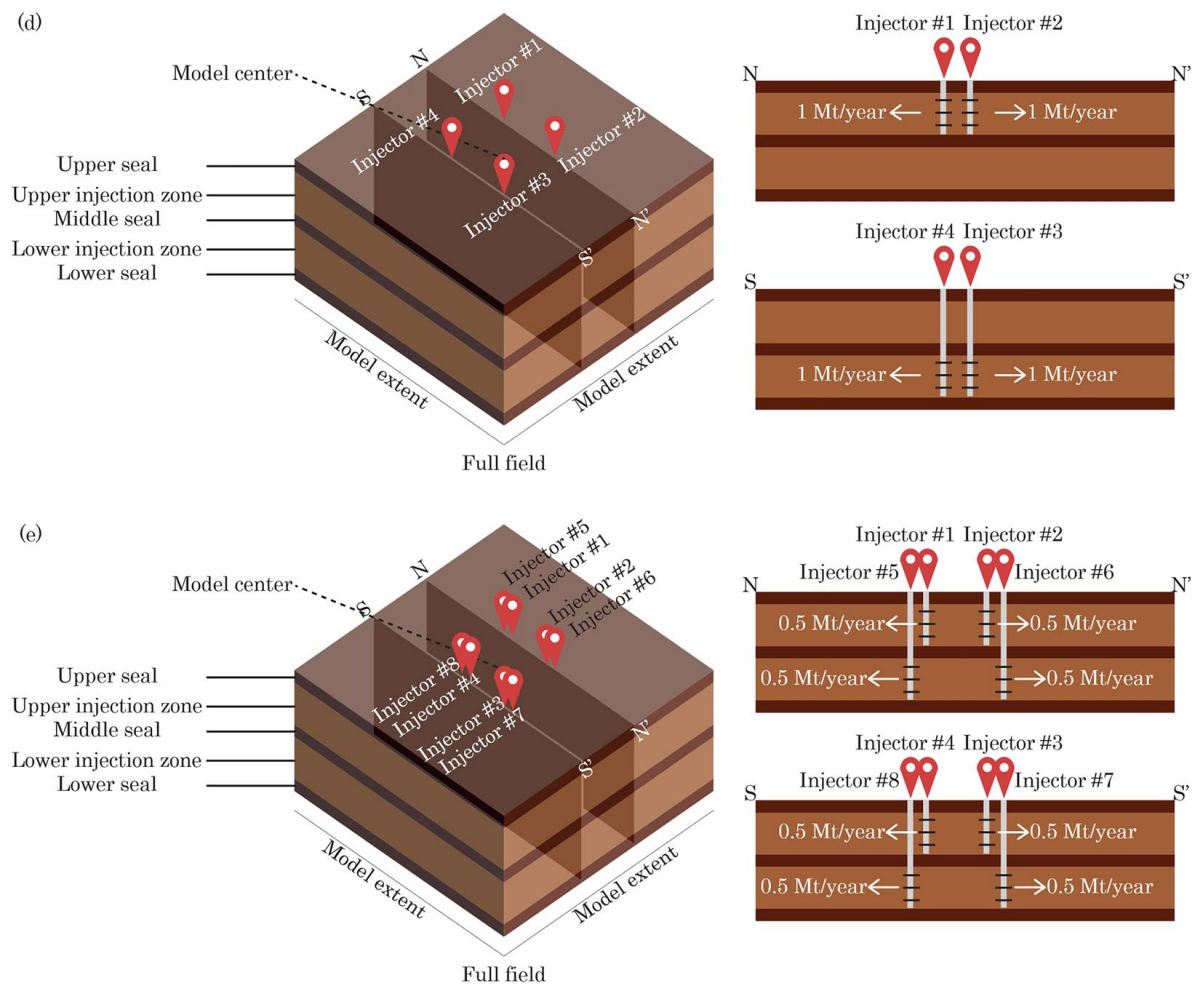
3-D schematics of modeling cases: Cases 1 through 5 in (**a**), (**b**), (**c**), (**d**), and (**e**), respectively, with cross sections (N-N’ and S-S’) for the multi-site cases. Figure is not scaled.

On the other hand, Cases 3 through 5 mimic the commercial-scale CO_2_ storage projects, in which the commercial-scale project unitization area consists of four storage sites to accommodate the target injection rates. The center of the model domain hosts the four storage sites that are placed on a five-spot pattern with a 5-km site spacing (Fig. 4), without any well or site at the center of the five-spot pattern. Cases 3 and 4 are identical from the aerial view; however, in Case 4, two of the sites inject CO_2_ into the lower injection zone rather than into the upper injection zone.

Each storage site may consist of one or two wells, depending on the number of target injection zones in each storage site (Fig. 4). Each injection well is assumed to be completed in a single injection zone only. When a particular storage site targets a single formation, this storage site hosts one injection well only with an injection rate of 1 Mt/year (before model segmentation). On the other hand, when a particular storage site targets a stacked sequence of two formations, this storage site hosts two injection wells (spaced only a few meters apart on the ground surface), each of which injects 0.5 Mt/year of CO_2_ (before model segmentation) into each respective target formation. Therefore, a storage site in this analysis is defined as a localized surface scope consisting of injection well(s) within a relatively narrow distance (i.e., a few meters only). As a result, in Cases 3 through 5, the total injection rate within the unitization area is 4 Mt/year of CO_2_. All injection wells are perforated throughout the entire thickness of each designated target formation.

In all modeling cases, CO_2_ is injected continuously (and concurrently when there are a few storage sites or wells) for 30 years. The injection constraint is constant CO_2_ injection rate. In addition, brine production was not evaluated as part of this work. Afterwards, the injection stops and is followed by 50 years of post-injection site care (PISC) stage (i.e., UIC Class VI well default). It is important to note that Fig. 4 is created as a schematic only, so that the dimensions shown are not based on a specific scale. Table 4 summarizes the correct model extent dimensions (i.e., aerial extent) in each case. The model mesh is laterally refined around the injection well to model the plume-scale processes and gradually coarser towards the model lateral boundaries. Given the large size of the reservoir model, this analysis leverages the parallel processing feature within TOUGH3 and runs all cases on NETL supercomputer Joule 2.0 to allow large memory requirements and higher-speed computation^58^.

By comparing the results from these modeling cases, this analysis aims to quantify the pressure buildup and CO_2_ plume extent and how the pressure buildup magnitude under the fracture pressure thresholds/constraints. A quantitative comparison among these cases allows a systematic analysis of the potential benefits of injecting CO_2_ into a vertically stacked sequence of saline formations, over injecting the same amount of CO_2_ into a single saline formation in addressing the challenges associated with the proximity among projects and the need for high injectivity.

## Results and analysis

In this section, the evolution of CO_2_ plume and pressure buildup of the five modeling cases as shown in Table 4 is covered consecutively. In both sections of CO_2_ plume and pressure buildup, Cases 1 and 2 are discussed first to establish the baseline on the extents of CO_2_ plume and pressure buildup when there is only one storage site in the model. Afterwards, the analysis will be followed by results from Cases 3 through 5 to provide insights into the effects of multi-site multi-well injection.

### CO_2_ plume extent

**Figure 5:**
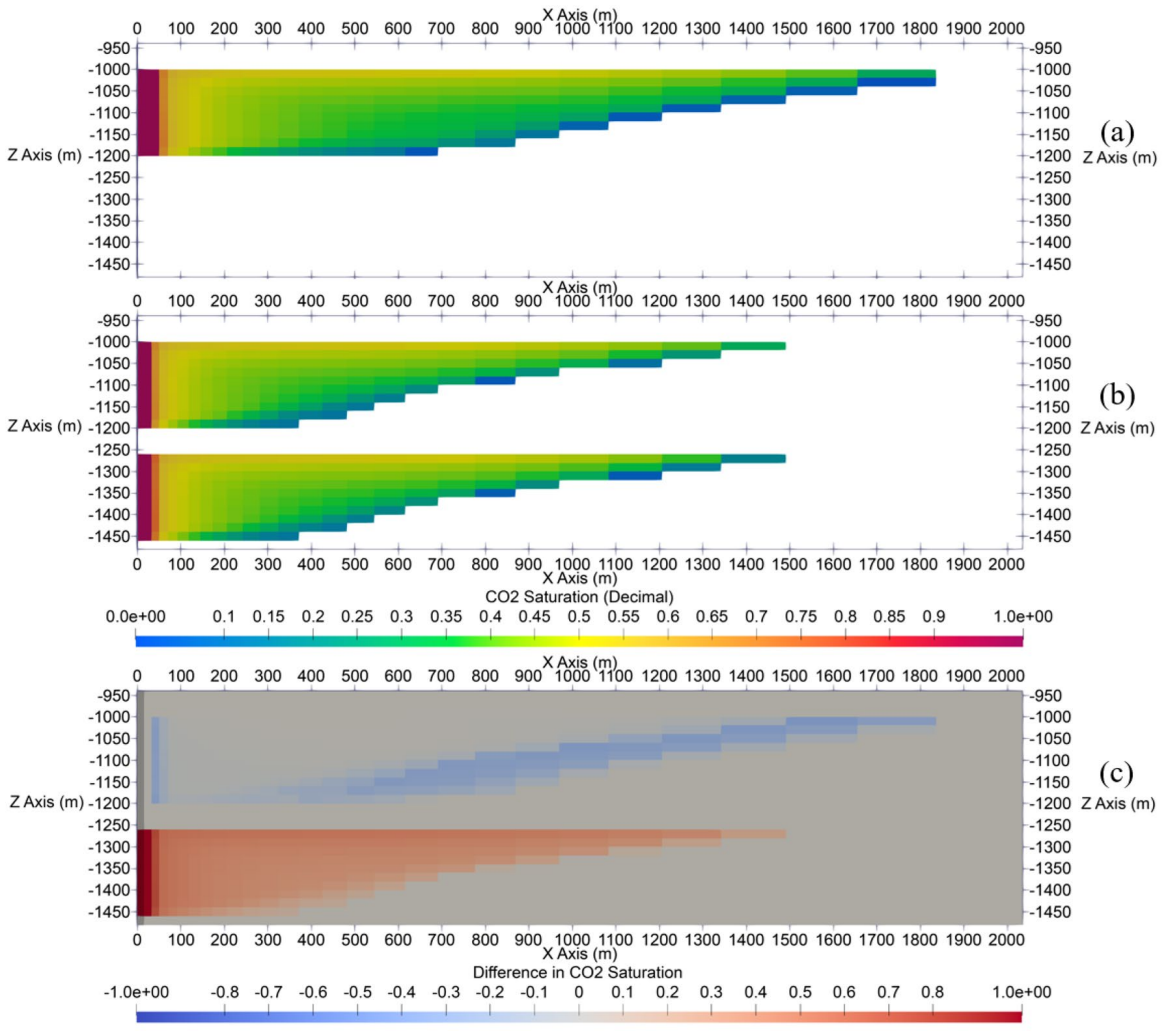
Side profile views of CO_2_ plumes (CO_2_ saturation range between 0 and 1) at the end of injection stage in single-site cases: (**a**) Case 1, (**b**) Case 2, and (**c**) the difference in CO_2_ saturation (CO_2_ saturation range between − 1 and 1) between two cases. The results show that the CO_2_ plume extent in upper formation in stacked Case 2 is less than in the Case 1.

The results shown for the single-site cases modeled, the radius of CO_2_ plume by the end of 30-year injection (Fig. 5) for Cases 1 and 2 is approximately 1.82 km and 1.5 km, respectively. Axis ticks in Fig. 5 are in unit of meters. Views in Fig. 5 show areas near the injection well only, rather than the entire model extent. As shown in Fig. 5, the CO_2_ plume demonstrates a gravity-override shape due to the buoyancy effect. Near the injection well, CO_2_ saturation is at its highest, while further away from the injection well, CO_2_ saturation tends to be lower toward the top of the storage formation.

Comparison between Fig. 5a,b shows that reducing the “per-well” injection rate from 1 Mt/yr in Case 1 to 0.5 Mt/yr in Case 2 (i.e., 50% reduction) does not proportionally decrease the radius of CO_2_ plume, since the radius only decreases from approximately 2 km in Case 1 to 1.5 km in Case 2 (i.e., 25% CO_2_ plume radius reduction).

For better visualization, Fig. 5c shows the difference in CO_2_ saturation between each grid block in Case 1 and Case 2 (i.e., CO_2_ saturation in Case 1 subtracted from that in Case 2).

**Figure 6:**
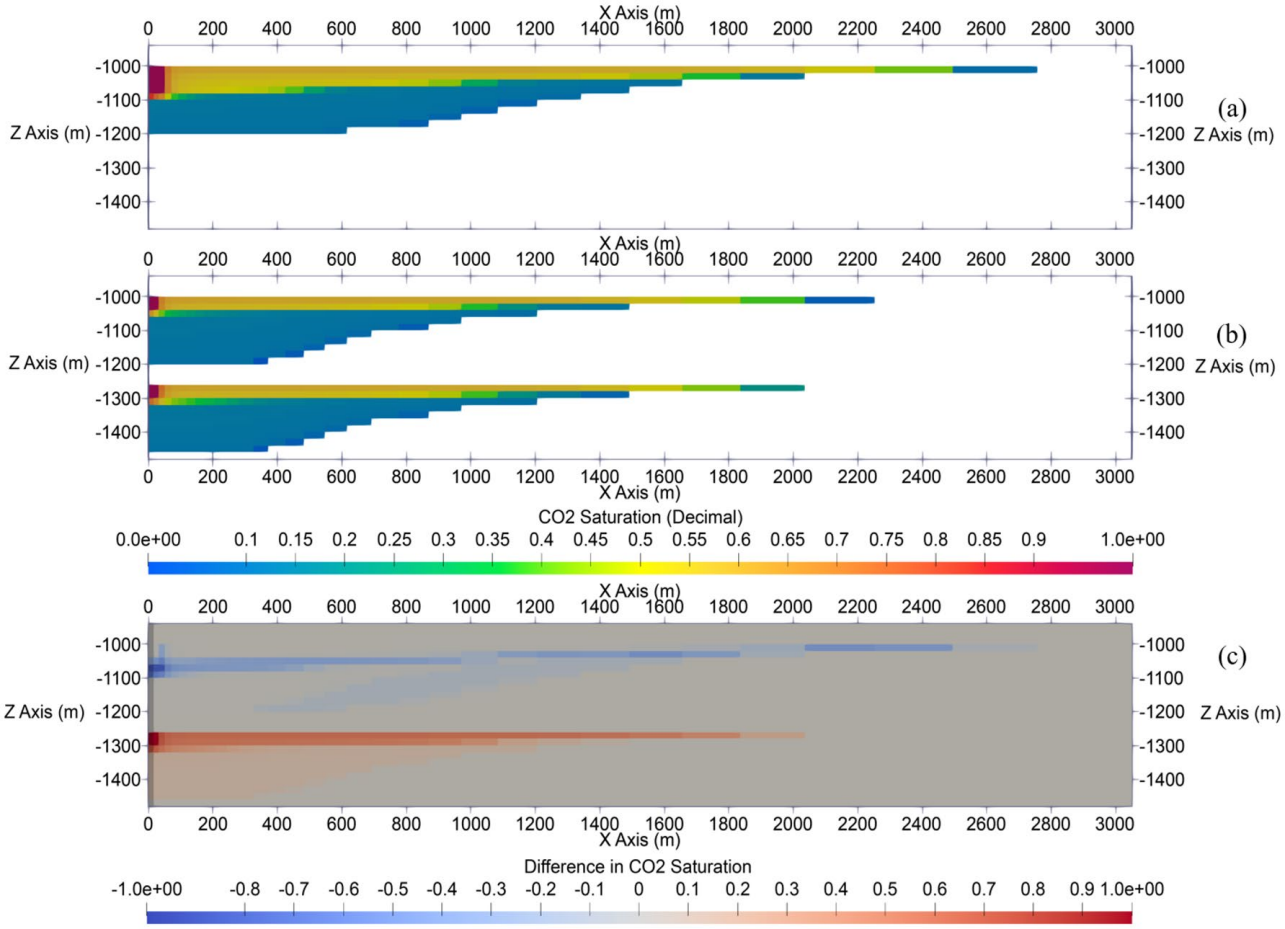
Side profile views of CO_2_ plumes (CO_2_ saturation range between 0 and 1) at the end of PISC stage in single-site cases: (**a**) Case 1, (**b**) Case 2, and (**c**) the difference (CO_2_ saturation range between − 1 and 1) in CO_2_ saturation between those cases. The results show that the CO_2_ plume extent in upper formation in stacked Case 2 is less than in the Case 1.

CO_2_ plume radius continues to increase after the end of injection due to the density contrast between CO_2_ and in-situ brine. CO_2_ tends to migrate from the previously occupied pores upwards in the storage formation and leaves these previously occupied lower zones with residual CO_2_ saturation, which represents the portion of CO_2_ storage volume trapped through the residual trapping mechanism. As shown in Fig. 6, the radius of CO_2_ plume by the end of 50-year PISC is approximately 2.75 and 2.25 km in Cases 1 and 2, respectively. The increasing radius of CO_2_ plume during PISC further highlights the importance of careful monitoring and modeling of site-specific storage projects.

**Figure 7:**
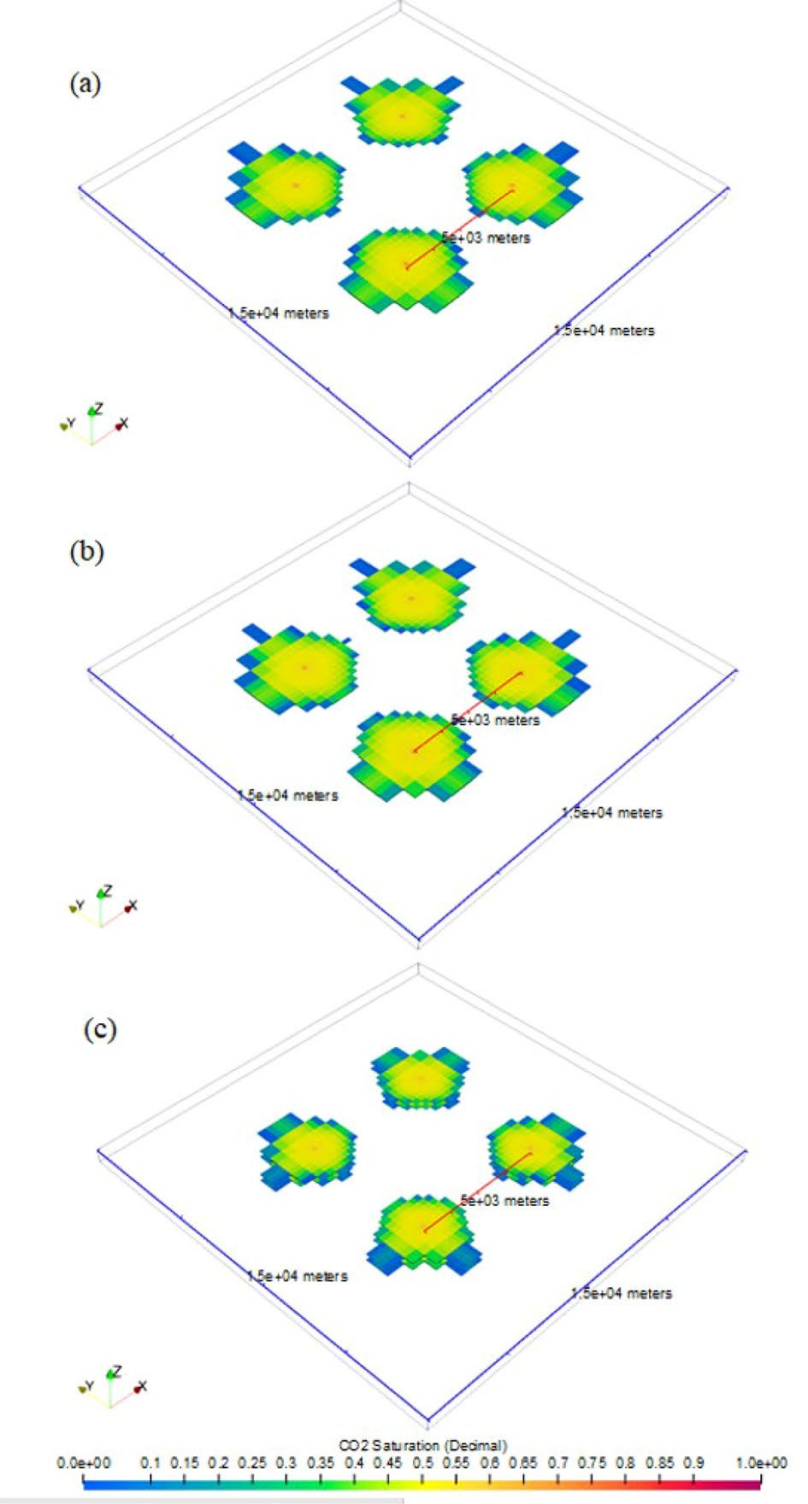
CO_2_ plumes at the end of injection stage in multi-site cases: (**a**) Case 3, (**b**) Case 4, and (**c**) Case 5.

**Figure 8:**
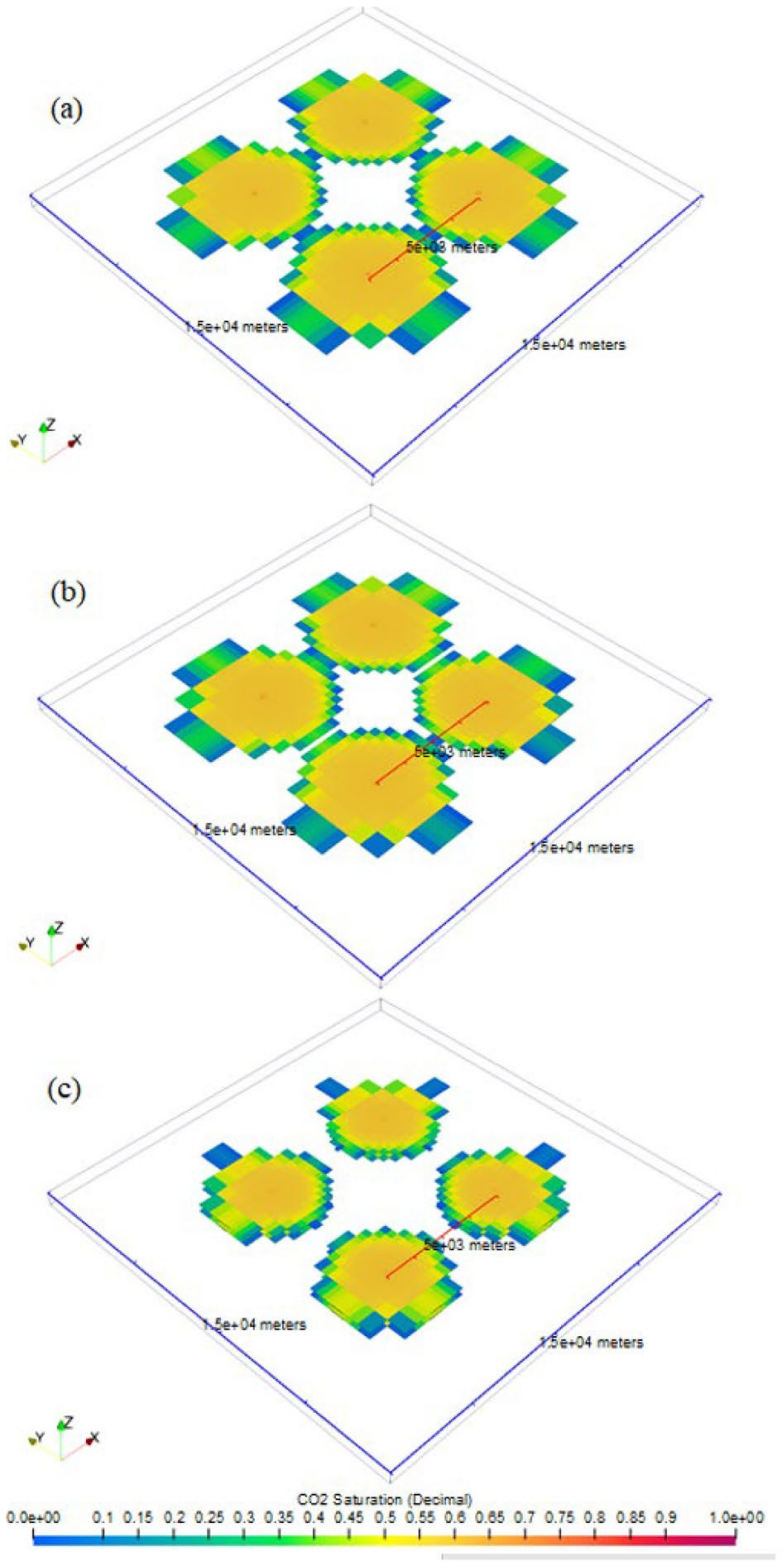
CO_2_ plumes at the end of PISC stage in multi-site cases: (**a**) Case 3, (**b**) Case 4, and (**c**) Case 5.

Multi-site cases demonstrate a site spacing of 5 km in Cases 3, 4, and 5. Figures 7 and 8 show the extent of CO_2_ plume for multi-site cases by the end of injection and PISC, respectively in top view. Figure 7 illustrates that by the end of 30-year injection, none of the multi-site cases indicates any CO_2_ plume commingling among the different storage sites. However, the radius of the CO_2_ plume continues to increase during PISC as shown the result of which appears to cause CO_2_ plume commingling after 50-year PISC in Cases 3 (Fig. 8a) and 4 (Fig. 8b). In case of potential CO_2_ leakage, legal challenges could exist associated with CO_2_ accounting and liability among these projects in carrying out work to remediate the leakage. In this analysis, PISC is assumed to be completed 50 years after the injection stops. It is likely that the CO_2_ plume continues to slightly increase beyond the modeled timeframe. Therefore, the 5-km site spacing used in this analysis is intended for case-demonstration purposes only, rather than proposing a universal optimum site spacing. The optimum site spacing should also be examined on a site-by-site basis from other standpoints, including storage zone thickness, porosity, injectivity, and maximum pressure buildup allowable per the EPA regulations, among many other considerations.

### Pressure buildup extent

**Figure 9:**
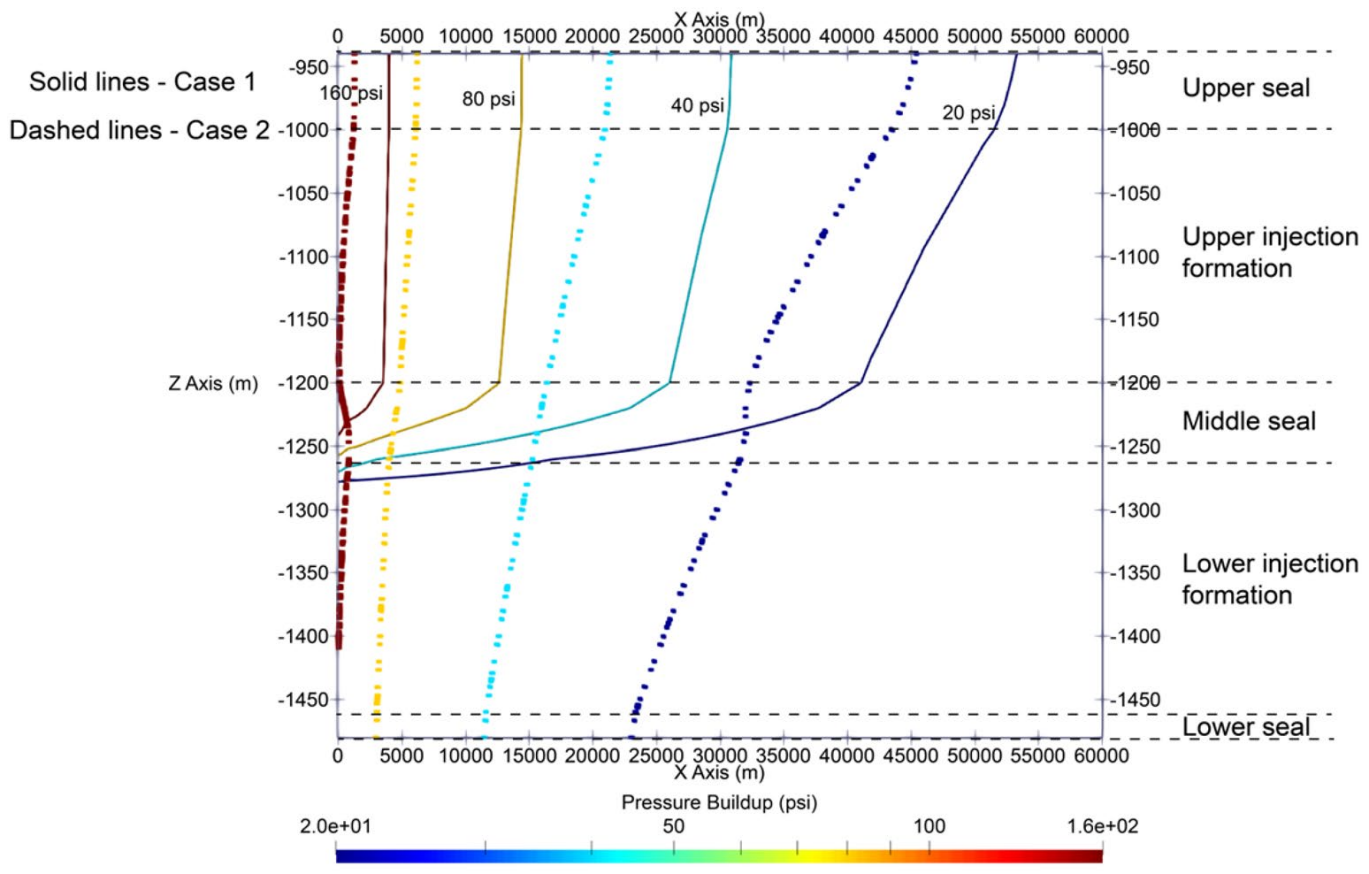
Side profile view of pressure buildup away from the injection well ($x=y=0$) at the end of injection stage in single-site cases: Case 1 (solid lines) and Case 2 (dashed lines).

The results show that the radius of pressure buildup front is in the range of tens of kilometers, depending on the pressure buildup magnitude defining the front while the radius of CO_2_ plume extends a mere 2.5 km or less. In this analysis, pressure buildup is calculated by subtracting the initial pressure at any grid block of interest from the pressure at that grid block at a certain time of interest as given by Eq. (1). Figure 9 presents a side profile view of contour lines showing the pressure buildup fronts of different pressure buildup magnitudes at the end of 30-year injection in the single-site cases. Axis ticks in Fig. 9 are in the unit of meters from wellbore. Figure 9 shows that in Case 1, the maximum radii of 20-psi and 160-psi pressure buildup are approximately 52 km and 4 km, respectively, while in Case 2, they are approximately 45 and 1 km, respectively. 20 psi is relatively small pressure buildup and almost arbitrarily selected in this analysis to trace the pressure-affected area. Such profiles are for a few pressure buildup magnitudes (i.e., 20, 40, 80, and 160 psi) for illustrative purposes only. Site-specific projects may be interested in other pressure buildup magnitudes, which can be higher than 20 psi.

Case 2 results in a consistently smaller radius of pressure buildup than Case 1, although both inject the same total volume of CO_2_. The reduction in the radius is more significant for smaller pressure buildup magnitudes. For example, for 20-psi and 160-psi pressure buildup, the reduction in the radius is approximately 8 km and 3 km, respectively. While from the lateral standpoint, distributing CO_2_ injection volume across the stacked sequence (Case 2) is shown to reduce the maximum pressure buildup radius, it consequently induces pressure buildup in the lower injection formation, which would have been minimum otherwise (i.e., if injection solely targets the upper injection zone as is modeled in Case 1). Figure 9 shows that in Case 1, pressure buildup propagates vertically across the low-permeability middle seal layer, although CO_2_ is permanently trapped in the upper injection formation. However, in Case 1, the vertical pressure propagation is minimum (i.e., even the 20-psi pressure buildup contour has not reached the bottom of the lower injection zone). Therefore, there is an exchange in terms of benefits offered between Cases 1 and 2 configurations; although both cases achieve the same injection volume, Case 1 prioritizes more on the preservation of the initial reservoir pressure of the lower injection zone for future or subsequent storage projects that target the lower injection zone, while Case 2 minimizes the magnitude of pressure buildup and interference among nearby storage sites sharing the same injection zones. Lastly, it is worth noting that the model domain in this analysis consists of layers shown in Fig. 9 only with all boundaries closed. It is expected that if the model domain includes additional layers above the upper seal and below the lower seal, the maximum pressure buildup radius in cases modeled in this analysis would decrease. The effect of model boundary condition was not evaluated as a part of this analysis.

The following discussion analyzes the range of ratios of pressure buildup and CO_2_ plume radius related to the estimation of pressure-affected area size. The radius ratio is given by:

4 $$\alpha \left(\Delta P,t\right)=\frac{{r}_{\Delta P}\left(\Delta P,t\right)}{{r}_{{\text{CO}}_{2}}\left(t\right)}$$

where $\alpha \left(\Delta P,t\right)$ is the ratio of the pressure buildup front radius of magnitude $\Delta P$ psi from the injection well to the CO_2_ plume radius at time $t$, ${r}_{\Delta P}\left(\Delta P,t\right)$ is the radius (km) of $\Delta P$-psi pressure buildup at the instant $t$ considered, and ${r}_{{\text{CO}}_{2}}\left(t\right)$ is the radius (km) of CO_2_ plume at the instant $t$ considered. It can also be called CO_2_-to-pressure-buildup multiplier. By the end of injection, this multiplier can be a useful parameter because it can help provide storage operators with a quick method to obtain a first order estimate of the pressure-affected area size delineated by pressure buildup given a CO_2_ plume area estimated from monitoring techniques. The pressure-affected area size influences CO_2_ storage costs because it includes costs associated with the number of monitoring wells that need to be deployed for a given commercial-scale project unitization area and the area of land in which operators need to carry out subsurface characterizations. Therefore, this multiplier could be a convenient parameter to help estimate the storage costs early in the project life before the pressure buildup has even reached its anticipated maximum radius throughout the project lifetime.

**Figure 10:**
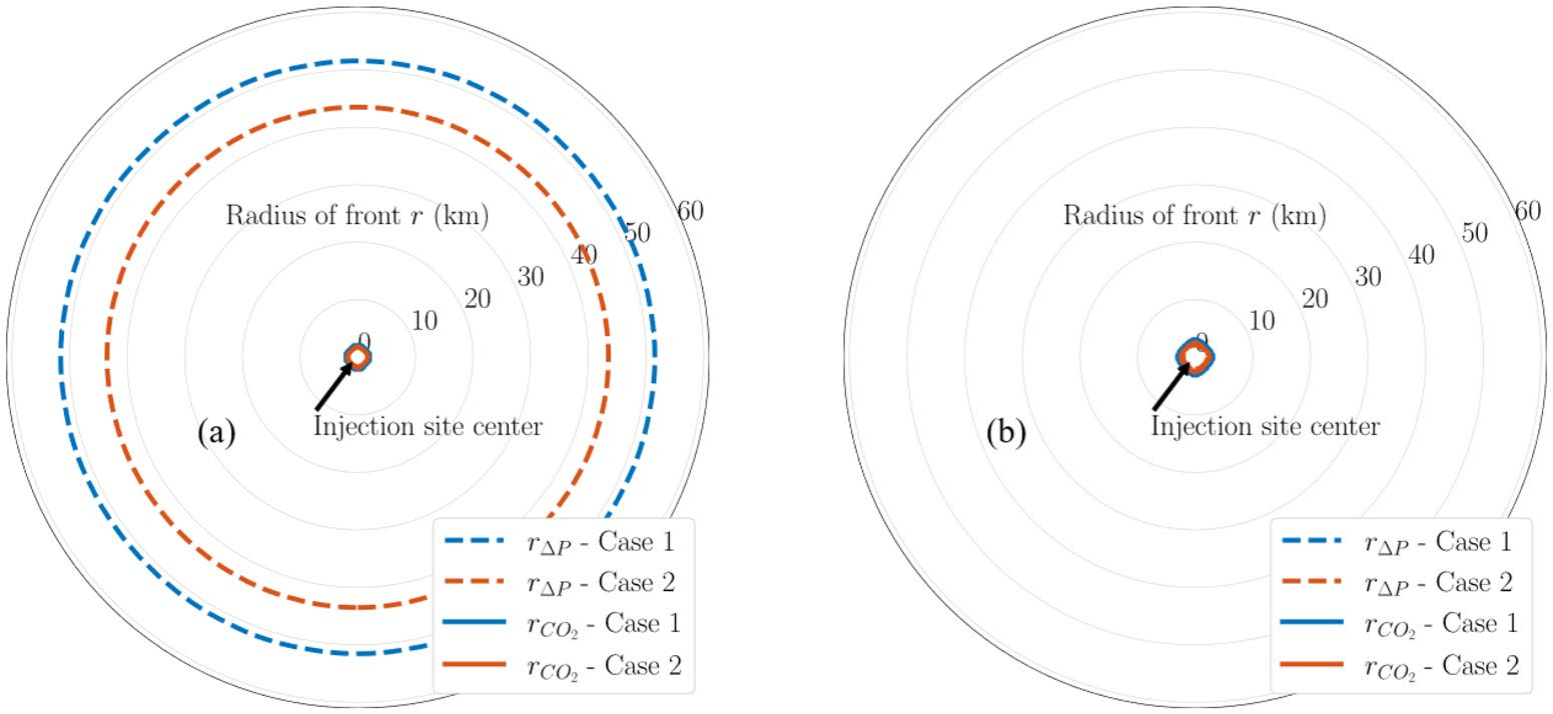
Map of 20-psi pressure buildup fronts and CO_2_ fronts in Cases 1 and 2 at (**a**) end of injection and (**b**) end of PISC stage. Radius labels are distances from the injection well.

**Figure 11:**
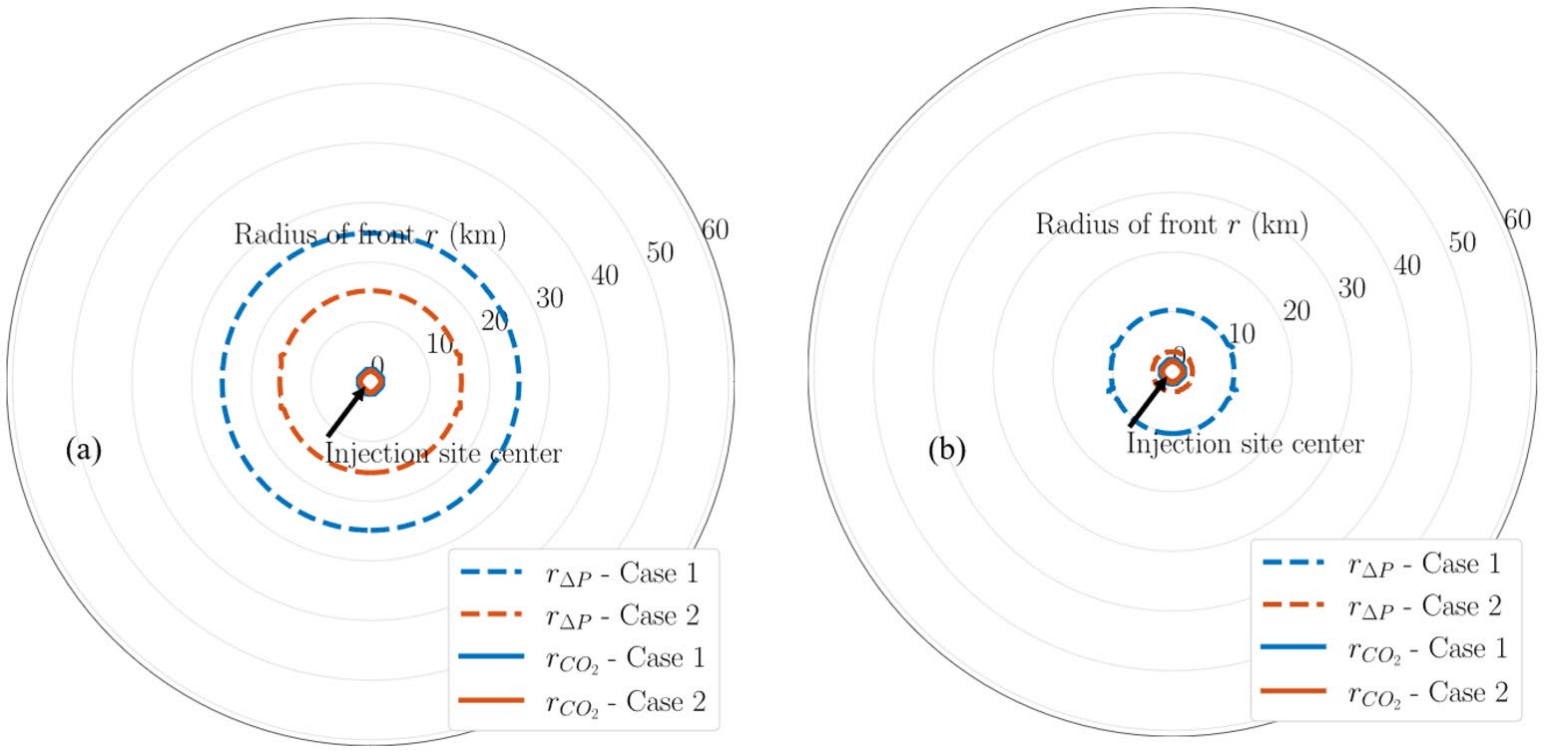
Map of pressure buildup fronts and CO_2_ fronts in Cases 1 and 2 at end of injection for (**a**) 50-psi; (**b**) 100-psi pressure buildup. Radius labels are distances from the injection well.

Figures 10 and 11 show the pressure buildup radius of varying magnitudes (i.e., 20, 50, and 100 psi) of the same modeling cases. Figure 10 presents a comparison in the aerial extent of 20-psi pressure buildup and CO_2_ fronts between Cases 1 and 2. The injection site center is located at the center of the diagrams. The radius labels are in the unit of kilometers. 20 psi is relatively small pressure buildup, which can be lower than the magnitude that site-specific projects are interested in. Therefore, 20-psi pressure buildup is discussed for illustration purposes only. The pressure buildup is taken at the depth of interface between the upper seal and upper injection zone. As shown in Fig. 10a, by the end of 30-year injection, the multiplier for 20-psi pressure buildup is approximately 25 and 22 for Cases 1 and 2, respectively.

As shown in Fig. 10b, by the end of 50-year PISC, most of the lingering pressure buildup is less than 20 psi since the 20-psi contours disappear. Figure 11 presents a comparison in the aerial extent of 50-psi and 100-psi pressure buildup and CO_2_ fronts between Cases 1 and 2 at the end of injection. These larger pressure buildup magnitudes no longer exist by the end of PISC due to pressure equilibration, hence no figures shown for end-of-PISC time slice for them.

Given reservoir and injection parameters, it is reasonable to obtain the large multipliers, particularly since 20 psi is a relatively low-pressure buildup magnitude. However, this multiplier will decrease as soon as the injection stops. As shown in Fig. 16b, the multiplier for 20-psi pressure buildup by the end of PISC decreases to approximately 1 for Cases 1 and 2 (i.e., similar radius between CO_2_ plume and 20-psi pressure buildup). This suggests that the benefit offered by Case 2 configuration (i.e., distributing the same CO_2_ injection volume into two injection zones) is time-dependent and primarily observed during the injection stage only.

**Figure 12:**
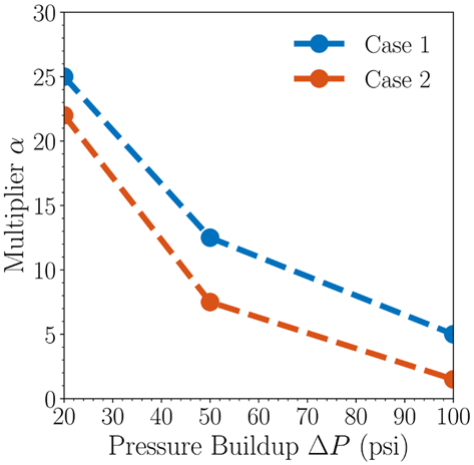
Maximum multipliers for CO_2_ plume to pressure buildup front radius at the end of injection for Cases 1 and 2.

Figure 11 shows that larger pressure buildup magnitudes correspond to a smaller CO_2_-to-pressure radius multiplier. As shown in Fig. 11a, the multiplier for 50-psi pressure buildup is approximately 12.5 and 7.5 for Cases 1 and 2, respectively. Likewise, as shown in Fig. 11b, the multiplier for 100-psi pressure buildup is approximately 5 and 1.5 for Cases 1 and 2, respectively. Figure 12 summarizes the multipliers at the end of injection for Cases 1 and 2. Figure 12 demonstrates that although larger pressure buildup consistently corresponds to a smaller multiplier, there does not seem to be a linear relationship in the multipliers for different pressure buildup magnitudes, which underscores that the multipliers depend on multiple interplaying factors, including the injection zone configurations (i.e., Case 1 compared to Case 2).

In the case of multiple storage sites (i.e., Cases 3 through 5), the radius of pressure buildup will increase correspondingly, since these cases assume a commercial-scale target CO_2_ storage volume (Table 4). Because the site spacing is 5 km only (Fig. 4), pressure interference is expected to occur rapidly among storage sites in the injection life and potentially increase the radius of commingled pressure buildup.

**Figure 13:**
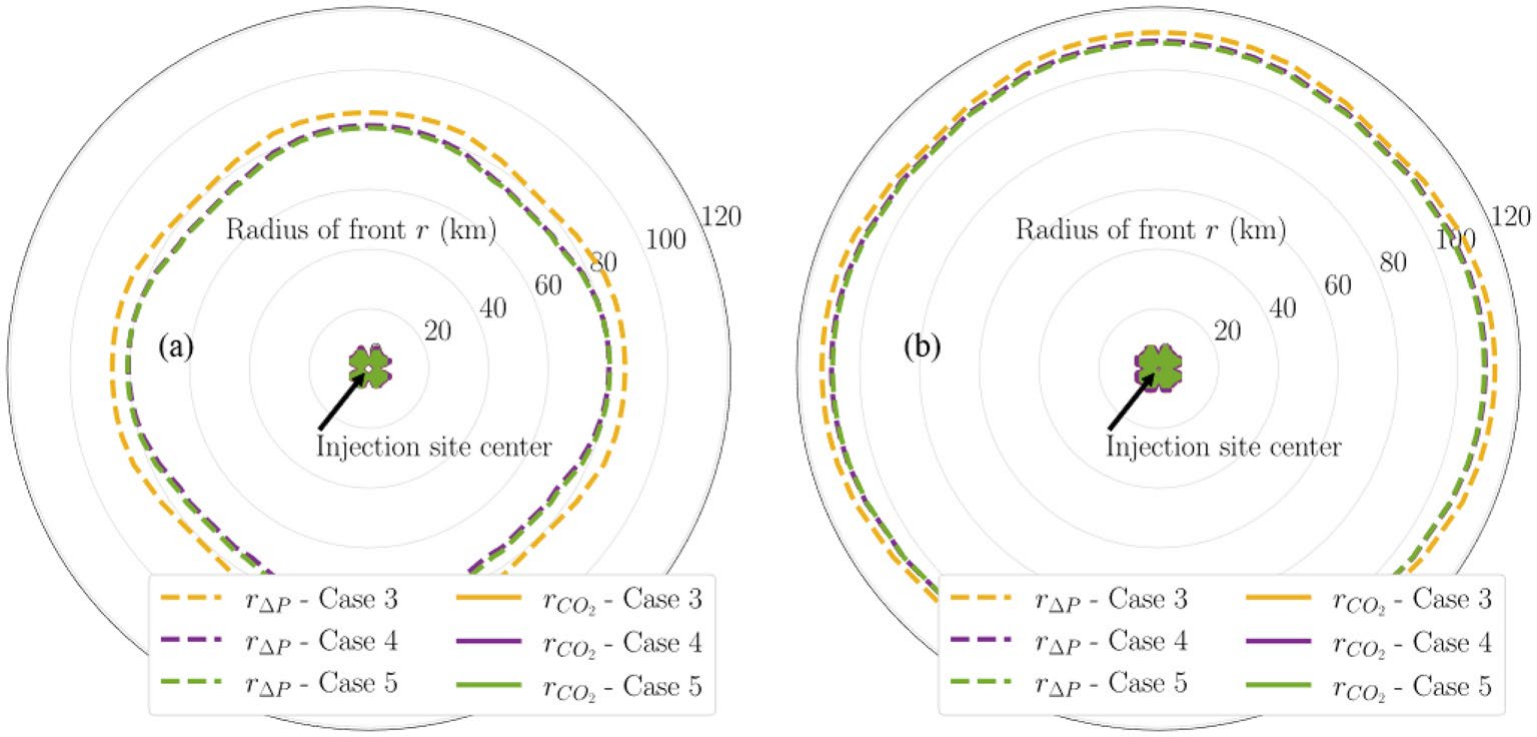
Map of 20-psi pressure buildup fronts and CO_2_ fronts in Cases 3 through 5 at (**a**) end of injection; (**b**) end of PISC stage. Radius labels are distances from the center.

**Figure 14:**
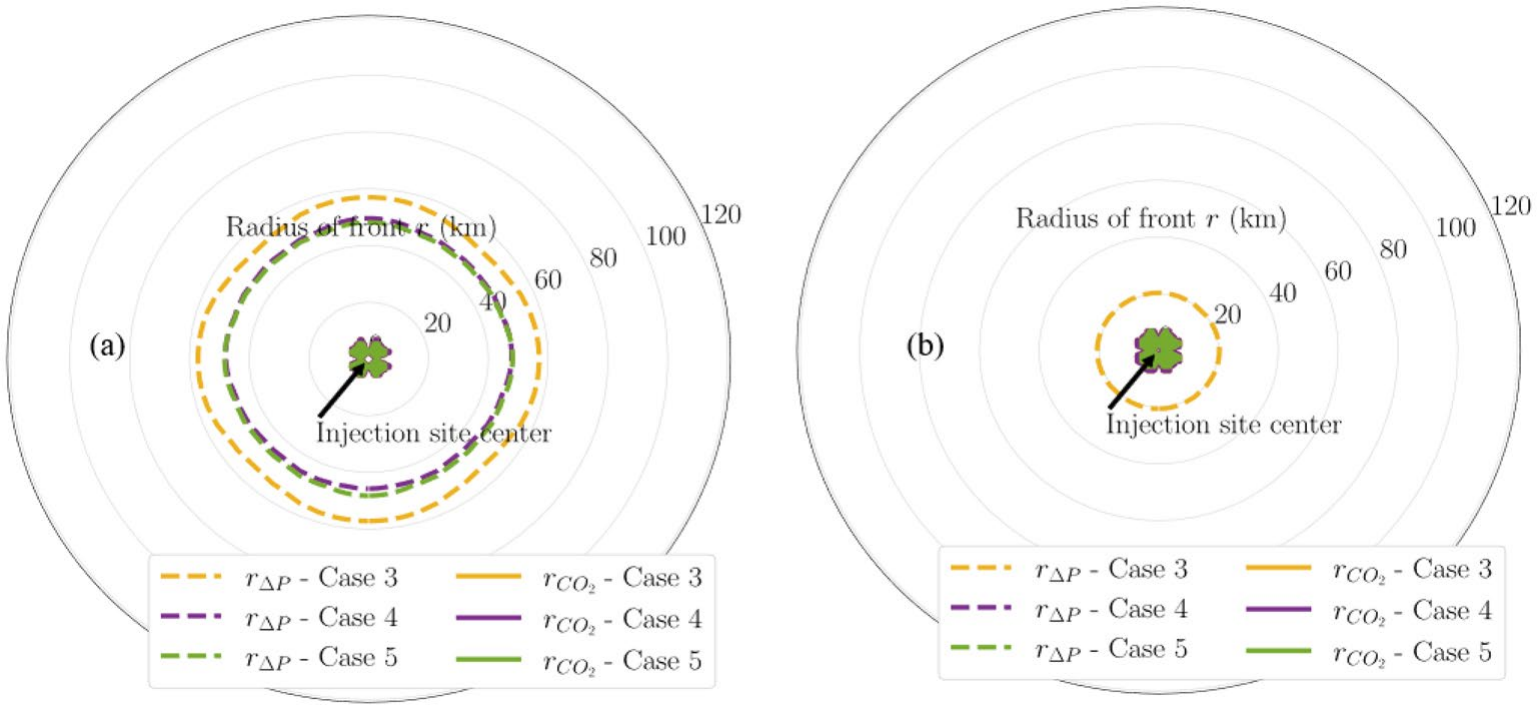
Map of 50-psi pressure buildup fronts and CO_2_ fronts in Cases 3 through 5 at (**a**) end of injection; (**b**) end of PISC stage. Radius labels are distances from the center.

**Figure 15:**
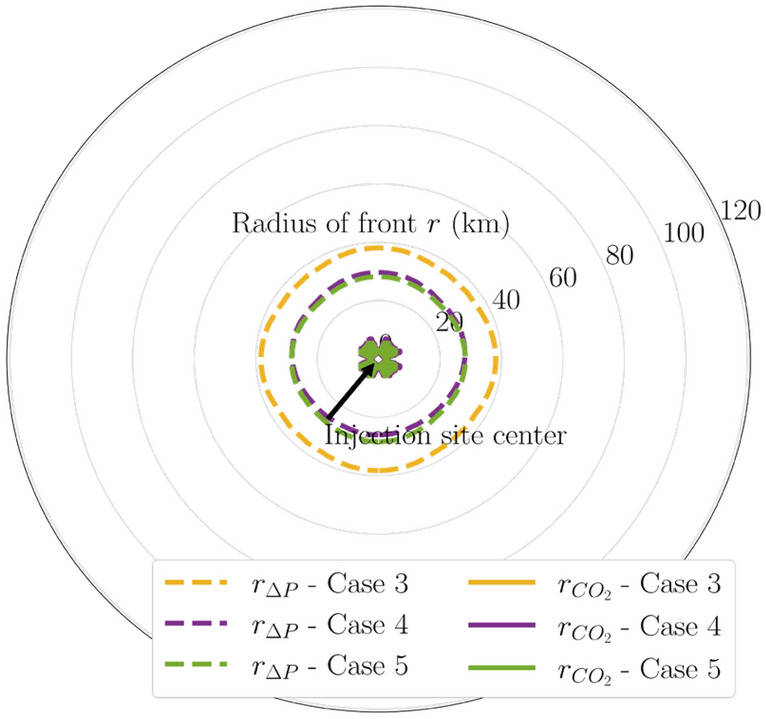
Map of 100-psi pressure buildup fronts and CO_2_ fronts in Cases 3 through 5 at end of injection. Radius labels are distances from the center.

Figures 13, 14, and 15 show the pressure buildup radius of varying magnitudes (i.e., 20, 50, and 100 psi) of the same modeling cases. Figure 13 presents a comparison in the aerial extent of 20-psi pressure buildup and CO_2_ fronts among the multi-site cases (i.e., Cases 3 through 5). The radius label range shown has increased from 60 km in the single-site cases (Fig. 10) to 120 km in the multi-site cases (Fig. 13) to capture the full extent of the fronts of interest in the multi-site cases. Figure 13a shows that by the end of 30-year injection, Case 3 results in the largest pressure buildup radius, because it targets a single injection formation only. In addition, Cases 4 and 5 are shown to have smaller pressure buildup radius than Case 3 but both cases have identical maximum pressure buildup radius, despite Case 5 having twice the number of injection wells as Case 4. This suggests that targeting the stacked sequence (i.e., Cases 4 and 5) rather than a single formation (i.e., Case 3) decreases the pressure buildup radius. However, deploying two wells rather than a single well at each storage site (i.e., Case 5 vs. 4) does not influence the pressure buildup radius. This means that the additional costs for constructing and operating the second injection well at each storage site might not be largely compensated because there seems to be no remarkable reduction in the pressure-affected area size from drilling the additional well. By the end of PISC (Fig. 13b), 20-psi pressure buildup is shown to propagate deeper into the formation due to the pressure equilibration but at a diminishing speed.

The benefits of distributing CO_2_ injection volume into the stacked sequence for decreasing the pressure buildup radius are consistently observed in other pressure buildup magnitudes as well. Figures 14 and 15 present the comparison for 50-psi and 100-psi pressure buildup for these multi-site cases, respectively. In both figures, injecting into the stacked sequence (i.e., Cases 4 and 5) results in a smaller pressure buildup radius than injecting into a single formation (i.e., Case 3).

**Figure 16:**
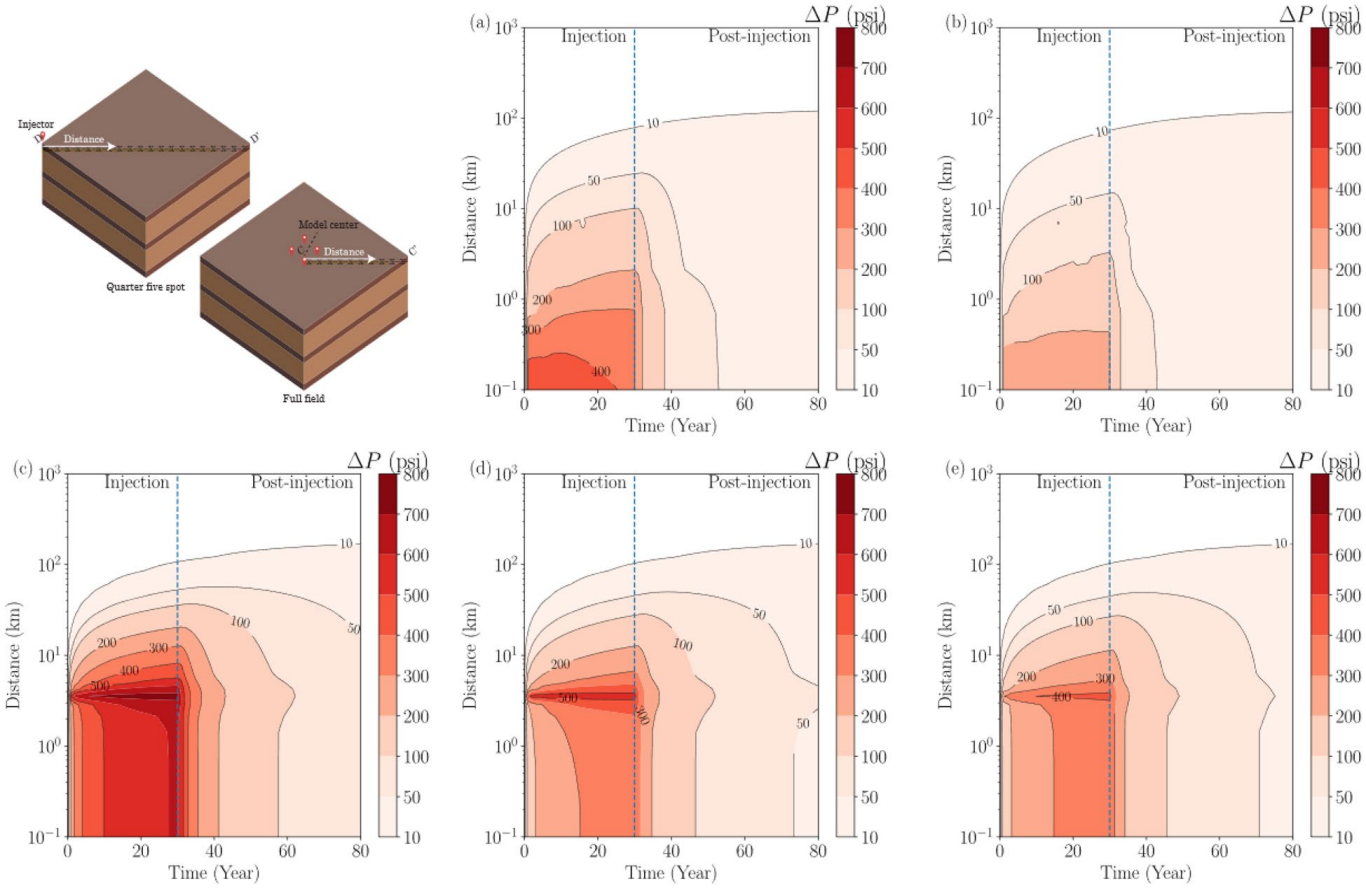
Evolution of pressure buildup along the monitoring points (X’s) on the line in the 3-D schematics during injection and PISC for all modeling cases: Cases 1 through 5 from (**a**) to (**e**), respectively. Distance is measured from point illustrated by white arrows in the 3-D schematics on the top-left corner: D-D’ for single-site cases and C-C’ for multi-site cases.

**Figure 17:**
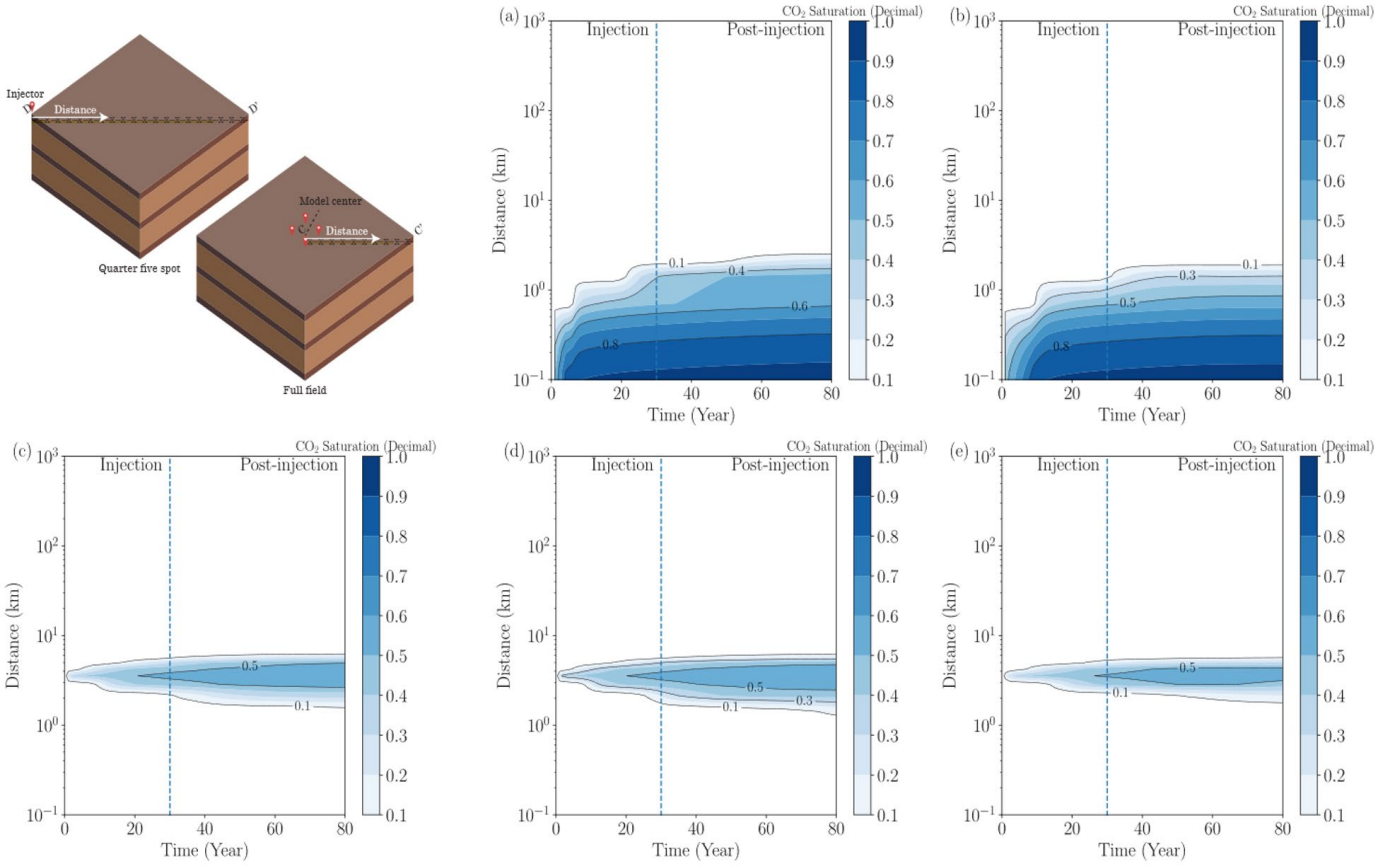
Evolution of CO_2_ saturation along the monitoring points (X’s) on the line in the 3-D schematics during injection and PISC for all modeling cases: Cases 1 through 5, respectively. Distance is measured from point illustrated by white arrows in the 3-D schematics: D-D’ for single-site cases and C-C’ for multi-site cases.

Besides the top views from Figs. 13, 14, and 15, cross-sectional comprehensive outlook of the spatiotemporal variability of pressure buildup and CO_2_ plume is presented in Figs. 16 and 17. In both figures, the distance is measured along the monitoring points illustrated in the 3-D schematics, depending on the model segmentation. The monitoring points are located at the depth of interface between the upper seal and the upper injection zone. The distance is presented in a logarithmic scale to show near-site and far-field monitoring data. The observations from both figures are in agreement with each other with what is shown in Figs. 13, 14, and 15 in top view.

### Fracture pressure threshold

Since distributing the injection volume across the stacked sequence is shown to decrease the radius of pressure buildup, it results in milder pressure interference among different storage sites. This could be a significant advantage of injecting into the stacked sequence in terms of avoiding the fracture pressure thresholds ($\gamma$) based on the EPA Class VI regulations as given by Eq. (2), which aims to mediate any potential large-scale pressure buildup to maintain the caprock integrity and prevent USDW contamination.

**Figure 18:**
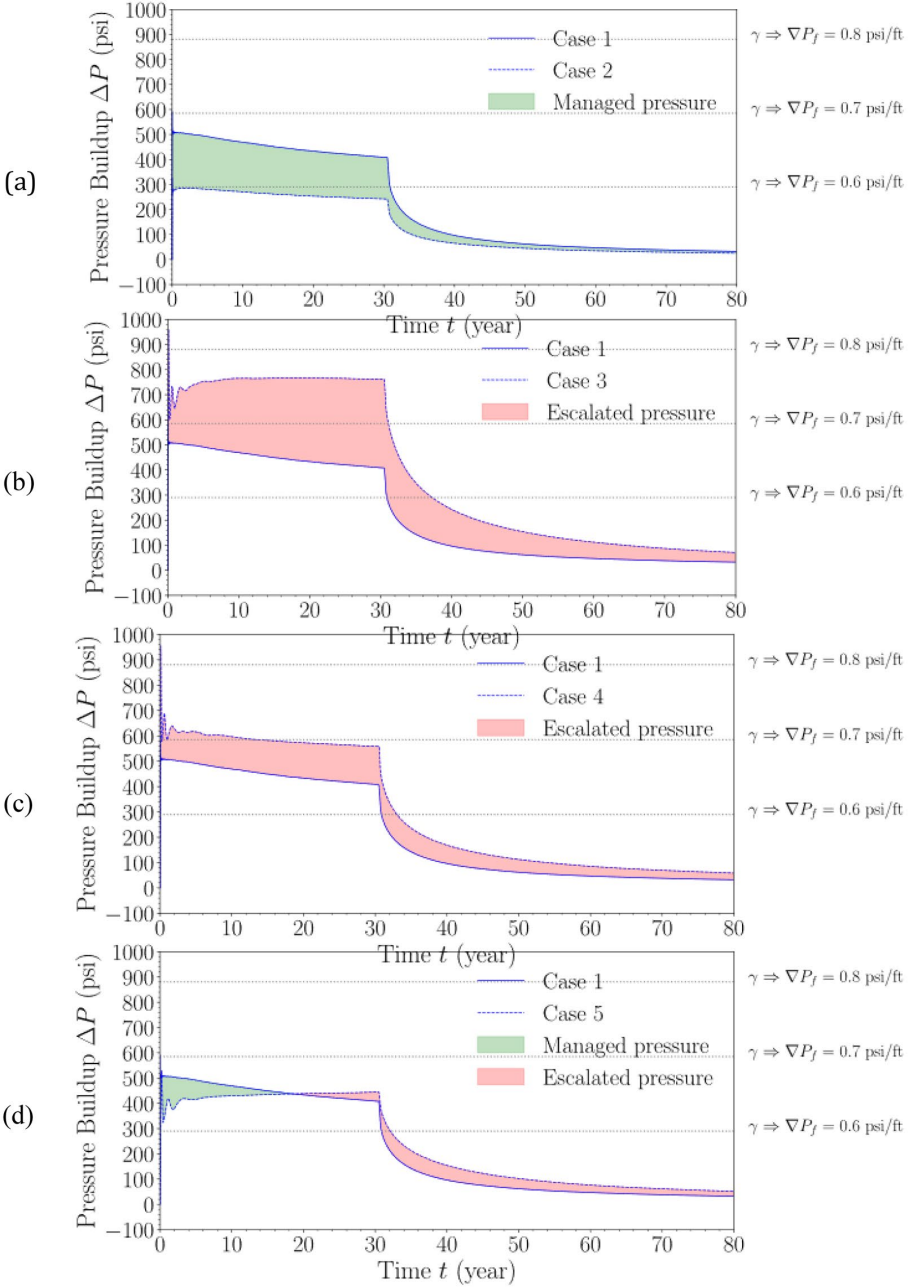
Pressure buildup ($\Delta P$) at the top of perforation at Injector #1 with varying fracture pressure thresholds in (**a**) Case 2, (**b**) Case 3, (**c**), Case 5 and (**d**) Case 5, respectively, with Case 1 considered as baseline. (**a**) Pressure buildup: Case 2 vs. Case 1 with various thresholds. (**b**) Pressure buildup: Case 3 vs. Case 1 with various thresholds. (**c**) Pressure buildup: Case 4 vs. Case 1 with various thresholds. (**d**) Pressure buildup: Case 5 vs. Case 1 with various thresholds.

Figure 18 shows the evolution of pressure buildup at the top of perforation of “Injector #1” (Fig. 4) in all cases modeled in this analysis. Since this analysis does not replicate any specific site, a range of fracture pressure gradients ($\nabla {P}_{f}$) is presented to provide insights into the likelihood of these modeled cases in approaching the fracture pressure thresholds for fracture gradients of 0.6, 0.7, and 0.8 psi/ft. In Fig. 18, Case 1 is treated as the baseline case, from which pressure escalation and/or management can be observed.

Among the commercial-scale cases modeled (i.e., Cases 3 through 5), Case 5 consistently shows the lowest pressure buildup at the injection well throughout the project duration. This shows that distributing CO_2_ injection volumes across the stacked sequence and having separate wells at each storage site (with each well injecting CO_2_ into different designated target formations in the stack), seems to be a favorable injection configuration for commercial-scale CO_2_ storage projects in terms of several considerations. For example, this configuration (Case 5) results in a smaller pressure buildup radius (and hence smaller pressure-affected area size) than injecting into a single formation (Case 3), while still achieving the same target injection volume. In fact, Fig. 18d demonstrates that Case 5 is shown to result in similar pressure buildup at the injection well as Case 1, although the total injected mass in Case 5 is four times larger than the mass in Case 1.

Comparison between Fig. 18c,d demonstrates that although Cases 4 and 5 result in a similar pressure-affected area size (Fig. 14), the pressure interference among nearby storage sites is managed more effectively in Case 5 by having two injection wells at each storage site. This could suggest that the additional costs of the drilling and operating the adjacent injection well in Case 5 could be compensated by the reduction in pressure buildup magnitude, which is a significant advantage in terms of avoiding the fracture pressure thresholds. This would allow operators to sustain the target CO_2_ injection rate for the project lifetime with fewer concerns of having to cut down the injection rate early in the injection stage to avoid the fracture pressure thresholds.

**Figure 19:**
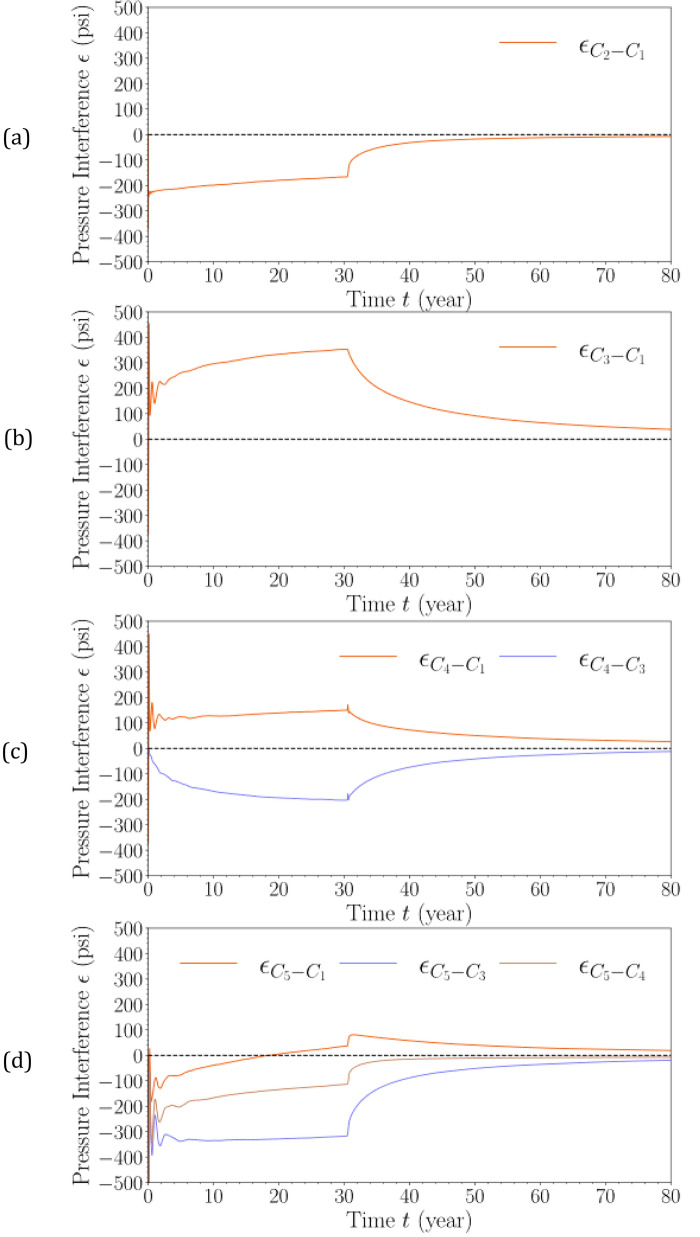
Pressure interference ($\epsilon$) at the top of perforation at Injector #1 in (**a**) Case 2, (**b**) Case 3, (**c**) Case 4, and (**d**) Case 5, respectively, with varying baseline cases. (**a**) Case 2 versus Case 1. (**b**) Case 3 versus Case 1. (**c**) Case 4 versus Cases 1 and 3. (**d**) Case 5 versus Cases 1, 3, and 4.

Figure 19 shows the pressure interference (${\epsilon }_{i-j}$) at the top of perforation at Injector #1 with variable baseline cases. As defined in Eq. (3), pressure buildup escalation (i.e., ${\epsilon }_{i-j}>0$) represents the resulting pressure interference in which the pressure buildup in Case $i$ at the given location and time considered is higher than the pressure buildup in Case $j$ at the corresponding location and time. On the other hand, negative pressure interference using this equation represents the pressure management extent (in psi) of deploying Case $i$ as opposed to Case $j$. Figure 19 demonstrates that pressure interference shows non-linear time-dependent behavior, especially during the early-time injection. Pressure interference is shown to depend on the selection of the baseline case. Since the primary interest of this analysis is to provide a one-to-one comparison in pressure buildup between stacked-sequence injection and single-formation injection, Case 1 is most frequently treated as the baseline case in Fig. 19. However, whenever potentially relevant, pressure interference calculation using other baseline cases (i.e., Cases 3 and 4) is also presented.

Figure 19 shows that stacked-sequence injection is an effective treatment for commercial-scale CO_2_ storage with narrowly spaced injection sites. When looking at single-site cases, there is approximately 200-psi reduction in pressure buildup throughout the injection stage with staked-sequence injection in single-site cases (i.e., Cases 2 vs. 1) (Fig. 19a); this translates to approximately 40% reduction in the pressure buildup in single-site cases. When looking at the commercial-scale cases, there is approximately 320-psi reduction in pressure buildup throughout the injection stage with stacked-sequence injection in multi-site cases (i.e., Cases 5 vs. 3) (Fig. 19d); this amounts to 42% reduction in the pressure buildup in commercial-scale cases. In all cases, as soon as the injection operation is complete, the pressure reduction benefit quickly diminishes. The pressure interference at the end of PISC is minimum because of pressure equilibration in the formations. However, this analysis evaluates a very specific geologic and injection situation; therefore, this analysis bears further investigation into other situations or specific basins. Future work also needs to combine the technical results on the subsurface dynamics of multi-site injection at each storage site with the economic implications of drilling the additional wells at each storage site.

## Discussions

Based on the results and analysis stated above, several points are worth further discussion:

For single injection site with the same amount of the injection such as 1 Mt/yr in Cases 1 and 2, the perforations in only one formation (upper in Case 1) and in both formations (upper and lower in Case 2) show the different CO_2_ plume extent and pressure movement. Such evidence adds the value for commercial-scale CO_2_ storage explorations based on the stacked formations. Besides, the results indicate the difference needs of monitoring and risk assessment for stacked storage as shown in the CO_2_ plume extent regarding to the area of review. The case with perforations in both formation (Case 2) is smaller than the one in upper the formation (Case 1). The similar observations for pressure buildup also illustrate the agreement even though the radius of the Case 2 is less than Case 1 as shown in Fig. 9. Moreover, based on the radius ratio definition, it demonstrates that there seems to be no linear relationship in the multipliers for different pressure buildup magnitude as shown in Fig. 12. More sensitivity analysis with heterogeneity in the following studies will provide solid validation and certain correlations between injection rate and plume extent size for both end of injection and PISC stages due to fluid dynamics in the system.

Multi-site cases are complex especially the control of operations in the case studies. With additional wells and amount of CO_2_ injection for Cases 3 to 5, the plume extent and pressure buildup are analyzed in detail as shows in Figs. 7, 8, 9, 10, 11, 12, 13, 14, 15, 16, and 17. Within the same amount of the injection target from each well, CO_2_ plume extent after 30 years injection shows no interference but after PISC, cases 4 and 5 are connected. It means that for multi-site injection, the designs, perforations, and well placements play curial role of reservoir response, even after 30 years of injection and the period for PISC. Though pressure buildup is less than 20 psi after PISC, the plume for both sites still show overlap as shown in Figs. 7, 8, 9, 10, 11, 12, 13, 14, and 15.

Further analysis of the pressure buildups and comparisons demonstrate that the stacked multi-site storage can reduce over 40% of the pressure buildup from the baseline in Case 1 as presented in Figs. 18 and 19. This adds a lot of value for the pressure management and de-risked CO_2_ storage monitoring and assessment to ensure permeant retention, especially for the large-scale deployment. Moreover, because of the CO_2_ plume extent and pressure buildup reduction, monitoring requirement and area of review (AOR) are reduced as well, which result in significant cost savings for carbon storage. Furthermore, associated monitoring, reporting and verification (MRV) plan and design are benefited from such reduced footprint impacts. Ultimately, it friendly promotes ecosystem and environmental regulations. Again, sensitivity analysis with heterogeneity, mid-seal zone, boundary condition tuning, and uplift risk in formations are parts of the following studies which will offer systematical optimization design for multi-site CO_2_ storage and reservoir managements in such large scale in the future work.

## Conclusions

Commercial scale decarbonization through CCS may likely involve many CO_2_ storage projects located in close proximity, which could raise concerns over caprock integrity due to potential large-scale pressure buildup in storage formations. This analysis investigates the benefits of injecting CO_2_ into a stacked sequence of saline formations in alleviating those concerns and accommodating the need for large prospective CO_2_ storage resource and high injectivity for the deployment of CCS at the commercial scale. This analysis employs numerical reservoir simulation to perform a systematic one-to-one comparison between injecting into the stacked sequence and into a single formation. A quantitative discussion on the effect of various injection configurations on the extent of CO_2_ plume and pressure buildup is presented. A range of fracture pressure gradients is analyzed with its regards in making sure that the pressure buildup at the injection well remains below the fracture pressure thresholds per EPA Class VI well regulations.

This analysis demonstrates that injecting CO_2_ into the stacked sequence offers significant benefits in terms of basin pressure management. This injection configuration results in both smaller overall pressure buildup magnitudes and smaller footprint of the pressure-affected area than injecting into a single formation, despite both configurations achieving the same CO_2_ storage volume. Deploying separate wells at each storage site with each well targeting different formations is shown to offer a significant additional reduction in pressure buildup magnitude to avoid the fracture pressure thresholds. Collectively, this configuration can offer approximately 40% and 42% reduction in the pressure buildup for single-site and multi-site cases, respectively by comparison of Cases 4 and 5 to baseline Case 1.

This analysis provides insights into the required decision-making when considering multi-project deployment in a shared basin. Because this analysis evaluates a very specific geologic situation, well configuration, and CO_2_ injection rate, this exploratory analysis bears further investigations across other geologic situations.

## Data Availability

The datasets used and/or analyzed during the current study available from the corresponding author on reasonable request.
