# Author Correction: Basin-scale study of CO_2_ storage in stacked sequence of geological formations

**DOI:** 10.1038/s41598-024-73100-9

**Published:** 2024-09-24

**Authors:** Nur Wijaya, David Morgan, Derek Vikara, Timothy Grant, Luciane Cunha, Guoxiang Liu

**Affiliations:** 1 National Energy Technology Laboratory (NETL) Support Contractor, 626 Cochrans Mill Road, P.O. Box 10940, Pittsburgh, PA 15236 USA; 2 NETL, 626 Cochrans Mill Road, P.O. Box 10940, Pittsburgh, PA 15236 USA; 3 Repsol USA, 2455 Technology Forest Blvd, The Woodlands, TX 77381 USA

Correction to: *Scientific Reports*10.1038/s41598-024-66272-x, published online 12 August 2024

Luciane Cunha was omitted from the author list in the original version of this Article.

The Author Contributions section now reads:

 “N.W.: Conceptualization, Methodology, Formal analysis, Visualization, Investigation, Programming, Data curation, Resources, Writing—Original draft, Writing—Review and editing. D.M.: Conceptualization, Methodology, Formal analysis, Writing—Review and editing, Project administration, Funding acquisition. D.V.: Conceptualization, Formal analysis, Writing—Review and editing, Project administration, Supervision. T.G.: Conceptualization, Formal analysis, Writing—Review and editing, Project administration, Funding acquisition. L.C.: Conceptualization, Formal analysis, Writing—Review and editing, Project administration, Funding acquisition, Supervision. G.L.: review and editing, corresponding the manuscript and publishing.”

The original Article has been corrected.

